# Aerophilic Triphase Interface Tuned by Carbon Dots Driving Durable and Flexible Rechargeable Zn-Air Batteries

**DOI:** 10.1007/s40820-022-00994-3

**Published:** 2023-01-03

**Authors:** Kuixing Ding, Yu Ye, Jiugang Hu, Liming Zhao, Wei Jin, Jia Luo, Shan Cai, Baicheng Weng, Guoqiang Zou, Hongshuai Hou, Xiaobo Ji

**Affiliations:** 1 College of Chemistry and Chemical Engineering, Central South University, Changsha, 410083 People’s Republic of China; 2 College of Standardization, China Jiliang University, Hangzhou, 310018 People’s Republic of China; 3 School of Environmental Science and Engineering, Suzhou University of Science and Technology, Suzhou, 215009 People’s Republic of China

**Keywords:** Aerophilic triphase interface, Oxygen-rich active sites, O_2_ diffusion, Bifunctional oxygen catalyst, Flexible rechargeable Zn-air battery

## Abstract

Efficient bifunctional catalysts for oxygen reduction reaction (ORR) and oxygen evolution reaction (OER) are vital for rechargeable Zn-air batteries (ZABs). Herein, an oxygen-respirable sponge-like Co@C–O–Cs catalyst with oxygen-rich active sites was designed and constructed for both ORR and OER by a facile carbon dot-assisted strategy. The aerophilic triphase interface of Co@C–O–Cs cathode efficiently boosts oxygen diffusion and transfer. The theoretical calculations and experimental studies revealed that the Co–C–COC active sites can redistribute the local charge density and lower the reaction energy barrier. The Co@C–O–Cs catalyst displays superior bifunctional catalytic activities with a half-wave potential of 0.82 V for ORR and an ultralow overpotential of 294 mV at 10 mA cm^−2^ for OER. Moreover, it can drive the liquid ZABs with high peak power density (106.4 mW cm^−2^), specific capacity (720.7 mAh g^−1^), outstanding long-term cycle stability (over 750 cycles at 10 mA cm^−2^), and exhibits excellent feasibility in flexible all-solid-state ZABs. These findings provide new insights into the rational design of efficient bifunctional oxygen catalysts in rechargeable metal-air batteries.

**Supplementary Information:**

The online version contains supplementary material available at 10.1007/s40820-022-00994-3.

## Highlights

- Oxygen-respirable sponge was constructed by carbon dots-assisted synthesis strategy.
- The hydrophilicity and aerophilicity of Co@C–O–Cs active sites boost oxygen diffusion and the bifunctional oxygen reduction reaction and oxygen evolution reaction activities.
- Co@C–O–Cs-based Zn-air battery (ZAB) displays excellent specific capacity and stability, and the all-solid-state Co@C–O–Cs-based ZAB exhibits excellent flexibility.

**Supplementary Information:**

The online version contains supplementary material available at 10.1007/s40820-022-00994-3.

## Graphical abstract

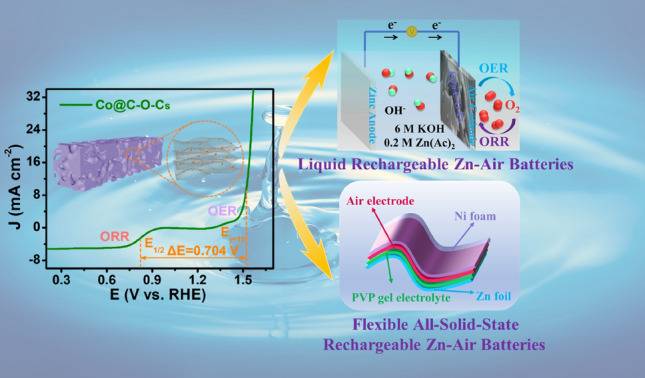

## Introduction

Nowadays, flexible electronics with portable and wearable characteristics have gradually attracted extensive attention in practical applications, such as foldable smartphones, roll-up displays, and smart bracelets [1–3]. To realize these novel energy storage devices, power sources with high energy density and excellent flexibility are essential [4, 5]. Flexible rechargeable zinc-air batteries (ZABs) are considered to be one of the most promising flexible/wearable energy storage devices owing to their high theoretical energy density (1086 Wh kg^−1^), low cost, remarkable safety, and environmental friendliness [6, 7]. Oxygen evolution reaction (OER) and oxygen reduction reaction (ORR) are essential for the charging and discharging processes, which determine the overall energy efficiency of ZABs. Since both OER and ORR undergo four electron transfer processes, their extremely slow proton coupling and electron transfer kinetics limit the energy conversion efficiency [8, 9]. So far, although precious Pt/RuO_2_ are outstanding catalysts for ORR/OER [10, 11], their high price, single catalytic function, and poor stability hinder their large-scale application [12]. Therefore, exploring cost-effective and non-noble-metal bifunctional oxygen catalysts for ORR and OER will greatly facilitate the development of flexible rechargeable ZABs.

In the past several years, various transition metal (Mn, Fe, Ni, and Co) catalysts, including transition metals [13, 14], oxides [15, 16], nitrides [17], phosphides [18], and chalcogenides [19, 20], have become extremely promising ORR and OER catalysts due to their abundant reserves and adjustable electronic states [21]. However, many transition metal catalysts have poor electrical conductivity and chemical durability, which seriously hinder their catalytic performance. It has been widely reported that the coupling between carbon materials and transition metal catalysts exhibits admirable activity and durability [22, 23]. In particular, the inevitable agglomeration of transition metal catalysts during high-temperature sintering can be effectively restrained through the confinement of carbon supports. Moreover, heteroatom (N, S, P, B, F, and Se) doping in carbon skeleton can activate the spin states and redistribute the charge of carbon atoms by adjusting the Fermi level of *sp*^2^ carbon [24], thereby improving the activities for ORR and OER. However, the influence of heteroatomic oxygen-containing species in the carbon-based catalysts on their electronic states and oxygen-catalytic activities is often ignored. Since the electronegativity of O (3.44) is greater than that of C atom (2.55), the doping of oxygen may regulate the electron delocalization of carbon skeleton and the adsorption of ORR/OER intermediates [25]. Moreover, oxygen doping can introduce abundant defection into the carbon skeleton as catalytic sites [26]. It is feasible to prepare bifunctional ORR/OER catalysts with high activity and excellent stability by coupling active metals and oxygen-doped carbon [27].

The performance of bifunctional catalysts not only relies on the intrinsic active sites but also highly depends on the utilization efficiency of the catalysts at the solid–liquid–gas three-phase interface. Several strategies have been reported to improve the three-phase interface environment, including surface/interface engineering [28], anion/cation modification [29], and all-in-one design [30], and so on. Wen et al. fabricated Co and Fe atoms encapsulated in the N,S-codoped hollow carbon spheres, and found that the CoFe-SNC catalyst exhibits excellent bifunctional ORR/OER activity and impressive Zn-air battery performance due to the continuous oxygen mass transfer at the three-phase contact region [31]. Tian et al. synthesized bifunctional B/N codoped carbon catalyst on carbon fibers for ORR/OER and found that the unique porous structure of BN-C/CF catalyst not only facilitates the exposure of more active sites, but also provides a specific channel for oxygen transport, making it exhibit remarkable capability for high-current and long-cycle Zn-air battery [32]. Zeng et al. designed NiCo_2_O_4_@N-OCNT composite catalysts by in situ encapsulation of NiCo_2_O_4_ nanoparticles on nitrogen–oxygen double-doped carbon nanotubes. The unique tubular structure of NiCo_2_O_4_@N-OCNT is beneficial to repel the formation of bubbles and alleviate the pinning effect of the bubbles at the three-phase interface [33]. These studies demonstrate that optimizing the surface/interface configuration of bifunctional catalysts is vital to sufficient exposure of active sites and rapid oxygen transport of air–cathode for flexible rechargeable ZABs.

Herein, guided by the density functional theory (DFT) calculations, an oxygen-respirable Co@C–O–Cs porous sponge with aerophilic triphase interface and oxygen-rich active sites was constructed with the assistance of carbon dots (CDs). Benefited from the high hydrophilicity and aerophilicity of Co@C–O–Cs at the three-phase interface, the O_2_ diffusion and mass transfer were accelerated, thus boosting the ORR/OER kinetics in alkaline solution. Theoretical calculations and electrochemical experiments revealed that the oxygen-rich active sites modulate the local charge density and lower the reaction energy barrier of carbon sponge, thus enhancing the ORR/OER activity. As expected, the Co@C–O–Cs catalyst delivers a decent half-wave potential of 0.82 V for ORR, and a low overpotential of 294 mV at 10 mA cm^−2^ for OER under an alkaline medium. The Co@C–O–Cs-based rechargeable liquid ZAB delivers superior performance with a high peak power density (106.4 mW cm^−2^), maximum specific capacity (720.7 mAh g^−1^), and outstanding long-term cycle stability (over 750 cycles) to Pt/C + RuO_2_-based ZAB. Furthermore, the flexible all-solid-state Co@C–O–Cs-based ZAB exhibits high power density and good flexibility at 0–180° bending.

## Experimental Section

### Materials

All the reagents were analytical pure and used without any further treatment. Acetaldehyde (40% water solution) was supplied by Shanghai McLean Biochemical Technology Co., Ltd. Sodium hydroxide was purchased from Tianjin Hengxing Chemical Reagent Co., Ltd. Hydrochloric acid and anhydrous ethanol were purchased from Chengdu Kelong Chemical Co., Ltd. Cobalt nitrate hexahydrate were provided by Sinopharm Chemical Reagent Co., Ltd. Nafion solution (5 wt% in alcohol and water) and ruthenium dioxide (RuO_2_, 99.9%) were provided from Sigma-Aldrich. A commercial Pt/C catalyst (20 wt% Pt on the black carbon) was purchased from Alfa Aesar. High-purity water (18.2 MΩ cm) was used for all the experimental processes.

### Material Synthesis

Carbon dots were synthesized by reacting sodium hydroxide with acetaldehyde at room temperature according to our previous work [34]. To obtain 3D porous sponge-like Co@C–O–C (denoted as Co@C–O–Cs), 1.5 g of cobalt nitrate and 2.0 g of CDs were dispersed in 50-mL absolute ethanol and magnetically stirred for 10 min to form a uniform dark brown solution. The solution was then placed in a round-bottomed flask and left to evaporate solvent completely, resulting in a dark brown powder. After loading into a ceramic boat, the dark brown powder was heated at 1000 °C under an Ar atmosphere for 2 h at a rate of 5 °C min^−1^, and then naturally cooled to 25 °C to obtain Co@C–O–Cs. The honeycomb-like Co@C–O–C (denoted as Co@C–O–Ch), vesicles-like Co@C–O–C (denoted as Co@C–O–Cv), and flake-like Co@C–O–C (denoted as Co@C–O–Cf) were also prepared by the same method except for the different calcination temperature (600, 800, 1200 °C). The Co@C–O–Cs was washed with 5 mol L^−1^ hydrochloric acid and distilled water to prepare the C–O–Cs control sample.

### Material Characterization

The microstructure of samples was characterized by using scanning electron microscopy (SEM, Magellan 400) and transmission electron microscopy (TEM, JEOL JEM 2100F). Power X-ray diffraction (XRD) were recorded on a Rigaku Ultimate Iv diffractometer with Cu Kα radiation (*λ* = 1.54 Å) operating at 40 kV and 250 mA. Raman spectroscopy was performed using a Raman spectrometer (Renishaw, inVia Reflex) with 532 nm-wavelength incident laser light. The Brunauer–Emmett–Teller (BET) specific surface area and pore size distribution were measured by a specific surface area and pore size analyzer (BET ASAP2460) with N_2_ as absorbate. The oxygen adsorption capacity of the prepared materials was determined from its O_2_ adsorption isotherms, which were obtained using a gas analyzer (Belsorp-Mini II, Japan). Inert atmosphere TG experiments were conducted on Netzsch-STA449F5 under the N_2_ atmosphere from 30 to 1200 °C with heating rate of 10 °C min^−1^. Oxidizing atmosphere TG experiments were conducted on Netzsch-STA449F5 under the oxygen atmosphere from 30 to 800 °C with heating rate of 10 °C min^−1^. X-ray photoelectron spectroscopy (XPS) was measured by Thermo Scientific EscaLab 250Xi. All XPS profiles were corrected using the C 1* s* line at 284.6 eV as an internal standard. C K-edge, Co L-edge, and O K-edge were measured at the beamline U19 of national synchrotron radiation laboratory (NSRL, Hefei) in the total electron yield mode by collecting the sample drain current under a vacuum better than 10^−7^ Pa. The Co K-edge were collected at BL11B station in Shanghai Synchrotron Radiation Facility and 4B9A beamline at the Beijing Synchrotron Radiation Facility. The acquired EXAFS data were extracted and processed according to standard procedures using the ATHENA module implemented in the IFEFFIT software packages. The *k*^3^-weighted EXAFS spectra were obtained by subtracting the post-edge background from the overall absorption and subsequently normalizing with respect to the edge-jump step.

### Electrochemical Measurements

All of the ORR and OER performance tests were performed at 25 ± 0.5 °C using a typical three-electrode system on a CHI 760E electrochemical workstation (Chenhua, Shanghai). A rotating disk electrode (RDE) with a glassy carbon disk (5.0 mm diameter) was typically used as the substrate for measuring ORR and OER. Ag/AgCl (saturated with 3.5 mol L^−1^ KCl) and a graphite rod were used as the reference and counter electrodes, respectively. And a 1.0 mol L^−1^ KOH solution was employed as the electrolyte. All potentials were converted to the reversible hydrogen electrode (RHE) scale according to the Nernst equation: *E*(RHE) = *E*(Ag/AgCl) + 0.198 V + 0.059 × pH. The RDE or rotating ring disk electrode (RRDE) coated with catalyst inks was used as the working electrode to investigate the kinetics behaviors of Co@C–O–Cs. To prepare the catalyst inks, 5.0 mg catalyst powders were dispersed into a mixed aqueous solution containing ethanol (490 µL), H_2_O (490 µL), and Nafion solution (5 wt%, 20 µL) under sonication for 1 h. Then, the obtained homogeneous catalyst inks were dropped onto RDE or RRDE and dried at 25 ± 0.5 °C to afford a mass loading capacity of about 0.26 mg cm^−2^. For comparison, the catalyst inks of both benchmark Pt/C and RuO_2_ with carbon black were also prepared with a loading amount of 0.26 mg cm^−2^. Prior to the ORR and OER catalytic activity experiments, the electrolyte was purged with high-purity O_2_ for 30 min to obtain an O_2_-saturated solution.

The ORR performance of the as-prepared samples was first investigated by CV in a N_2_/O_2_-saturated 1.0 mol L^−1^ KOH solution at 25 ± 0.5 °C at a sweep rate of 50 mV s^−1^. LSV experiments were performed in a O_2_-saturated 1.0 mol L^−1^ KOH solution at different rotating speeds (400–1600 rpm) with a scan rate of 5 mV s^−1^. For RDE measurements, the K–L Eqs. (1–3) were used to calculate the kinetic current density (*J*_k_) and transferred electron number (*n*):

1 $$\frac{1}{J}=\frac{1}{{J}_{\mathrm{k}}} +\frac{1}{{\mathrm{B}\omega }^{1/2}}$$

2 $$B=0.2nF{D}_{0}^{2/3}{\nu }^{-1/6}{C}_{0}$$

3 $${J}_{\mathrm{k}}=\mathrm{nFk}{C}_{0}$$

where *ω* is the angular velocity of disk (*ω* = 2π*N*, *N* is the linear rotation speed); *F* is the Faraday constant (*F* = 96,485 C mol^−1^); *C*_0_ is the bulk concentration of O_2_ (1.2 × 10^−6^ mol cm^−3^); *D*_0_ is the diffusion coefficient of O_2_ (1.9 × 10^−5^ cm^2^ s^−1^ for 1.0 mol L^−1^ KOH); and ν is the kinetic viscosity of the electrolyte (0.01 cm^2^ s^−1^). For RRDE measurements, the percentage of intermediate production of HO_2_^−^ (%HO_2_^−^, which indicates the selectivity to 2e^−^ pathway) and electron number transferred per oxygen molecule (*n*) were determined were determined as follows:

4 $$\mathrm{\%H}{\mathrm{O}}_{2}^{-}=\frac{200{I}_{r}}{N{I}_{d}+{I}_{r}}$$

5 $$n=\frac{4N{I}_{d}}{N{I}_{d}+{I}_{r}}$$

where *I*_d_ and *I*_r_ represent the disk and ring currents, respectively; and *N* is the current collection efficiency of the Pt ring, which was determined to be 0.37 [35]. The stability of the samples was examined by collecting current–time (*i*-*t*) chronoamperometric responses at 0.67 V (vs. RHE) in 1.0 mol L^−1^ KOH for 12,000 s.

For OER measurements, the LSV scan was conducted from 1.2 to 1.8 V (vs. RHE) at a scan rate of 5 mV s^−1^ and corrected by 95% *iR* compensation to compare the performance of the catalysts, where* i* is the measured current and *R* is the compensated resistance between the working and reference electrodes. The overpotential was calculated using *η* = *E* (vs. RHE) − 1.23 V. The OER stability of the samples was examined based on *i*-*t* chronoamperometric responses at 1.8 V (*vs.* RHE) for 12,000 s. The ECSAs of the catalysts were compared using the double-layer capacitance (*C*_dl_) in the non-Faradaic region from 1.20 to 1.30 V *vs.* RHE. The scanning rates were set at 10, 20, 40, 60, 80, and 100 mV s^−1^. EIS experiments were performed using a Zahner electrochemical analyzer (Zennium, Germany) in the frequency range of 100 kHz to 10 mHz. The applied potential and excitation amplitude were set to 1.6 V (*vs.* RHE) and 5 mV, respectively.

### Assembly and Measurements of Liquid Zn-air Battery

To evaluate the performance of liquid Zn-air batteries, a home-made two-electrode device was constructed. The air electrode was made of nickel foam with a gas diffusion layer on the air-facing side and a catalyst layer on the electrolyte-facing side. The gas diffusion layer with an effective area of 1.0 cm^2^ allowed O_2_ diffusion from ambient air to the catalyst sites. The catalyst layer was made by drop-casting the catalyst ink onto the nickel foam with a loading capacity of 6.0 mg cm^−2^ for all the catalysts. The anode was a polished Zn plate with a thickness of 1.0 mm (99.99% purity). The electrolyte solution contained 6.0 mol L^−1^ KOH and 0.2 mol L^−1^ Zn(Ac)_2_. A rechargeable battery fabricated using a mixture of commercial Pt/C (20%) and RuO_2_ (99.95%) with a mass ratio of 1:1 was used for comparison. All battery performance tests were performed under an ambient atmosphere. The charge–discharge cycling tests of the rechargeable liquid ZABs were performed at 10 mA cm^−2^ with 20 min per cycle (10 min for charging and 10 min for discharging) using the Land-CT2001A testing system.

### Assembly and Measurements of Flexible Solid-state Zn-air Battery

The gel polymer electrolyte (GPE) was firstly prepared as follows: 2.0 g of PVA powder was added into 20 mL of deionized water, followed by magnetically stirring for 1 h at 90 °C. After the solution was transformed into transparent state, 2 mL of 18.0 mol L^−1^ KOH solution containing 0.2 mol L^−1^ Zn(Ac)_2_ was added into the mixture. After stirred for 0.5 h, the gel was poured into a container and frozen at −20 °C for 2 h. The PVA gel electrolyte was obtained after thawing at room temperature. In a typical assembly of a flexible all-solid-state Zn-air battery, a clean Zn foil (0.08 mm thickness) was used as anode. The air electrode was made by dropping catalyst ink onto a flexible nickel foam substrate with a catalyst loading capacity of 1.5 mg cm^−2^. Then, the flexible solid-state Zn-air battery was assembled with air electrode and Zn foil placed on the two sides of the PVA gel electrolyte, and two pieces of acrylic tape were used to seal the device. The galvanostatic charge–discharge curves were recorded by chronopotentiometry at a current density of 1 mA cm^−2^ with 20 min per cycle (10 min charge and 10 min discharge).

### DFT Calculations

Computational calculations were performed based on DFT with generalized gradient approximations (GGA) [36, 37] implemented in the Vienna ab initio simulation package (VASP) [38, 39]. The *k* points in the Brillouin zone were sampled with 3 × 2 × 1 unit cell for the structural optimization, with four lines being used along the Brillouin zone for the self-consistent calculations. The vacuum layer was set to 15 Å filled along the *c* axis of the unit cell to avoid interactions between the periodic images. The pseudopotential generated in a projector augmented wave (PAW) [40] was used for the electron–ion interactions. In addition, the influence of spin polarization was considered in the calculation process. During geometric optimization, the bottom atomic layer was fixed, and all other atoms and adsorbates were fully relaxed until the maximum force on each atom was less than 0.02 eV Å^−1^. The total energy convergence criterion between every two electronic steps was set to 10^−5^ eV. All models were geometrically optimized prior to the single-point energy calculations.

Changes in the free energies of the ORR and OER were obtained using the DFT calculations. The ORR could proceed incompletely through a two-electron pathway and reduce O_2_ to H_2_O_2_, or completely progress via a four-electron route and reduce O_2_ to H_2_O; the latter was adopted herein because of its efficiency and desirability. Therefore, the 4e^−^ reaction mechanism about ORR could be presented as below:

6 $${\text{O}}_{{2}} \left( {\text{g}} \right) \, + {\text{ H}}_{{2}} {\text{O }}\left( {\text{l}} \right) + * + {\text{e}}^{ - } \leftrightarrow *{\text{OOH }} + {\text{ OH}}^{-}$$

7 $$*{\text{OOH}} + {\text{ e}}^{ - } \leftrightarrow *{\text{O}} + {\text{OH}}^{ - }$$

8 $${\text{O}}* + {\text{ H}}_{{2}} {\text{O}}\left( {\text{l}} \right) + {\text{e}}^{ - } \leftrightarrow *{\text{OH }} + {\text{ OH}}^{ - }$$

9 $$*{\text{OH }} + {\text{ e}}^{ - } \leftrightarrow * + {\text{OH}}^{ - }$$

where * refers to active sites; (l) and (g) stand for liquid and gas phases, respectively; and *O, *OH, and *OOH are the adsorbed intermediates. As a reverse reaction of ORR, the mechanism of OER is described as below:

10 $${\text{OH}}^{ - } + * \leftrightarrow *{\text{OH}} + {\text{e}}^{ - }$$

11 $$*{\text{OH}} + {\text{OH}}^{ - } \leftrightarrow *{\text{O}} + {\text{H}}_{{2}} {\text{O}}\left( {\text{l}} \right) + {\text{e}}^{ - }$$

12 $$*{\text{O}} + {\text{OH}}^{ - } \leftrightarrow *{\text{OOH}} + {\text{e}}^{ - }$$

13 $$*{\text{OOH}} + {\text{OH}}^{ - } \leftrightarrow * + {\text{O}}_{{2}} \left( {\text{g}} \right) + {\text{H}}_{{2}} {\text{O}}\left( {\text{l}} \right) + {\text{e}}^{ - }$$

The Gibbs free energy change Δ*G* of each elementary step was calculated as:

14 $$\Delta G = \Delta E + \Delta {\text{ZPE}} - T \cdot \Delta S + \Delta G_{U}$$

where *∆E* is the reaction energy; *∆*ZPE is the change of zero-point energy; *T* (298.15 K) is temperature; *∆S* is the difference in entropy. The zero-point energies were calculated from the vibration frequencies [41]. The entropies were taken from standard tables for gas-phase molecules [42]. Gas phase H_2_O at 0.035 bar and 300 K was used as the reference state because it equilibrated with liquid water at this situation.

15 $$\Delta G_{{*{\text{O}}}} = \, \Delta G\left[ {{\text{H}}_{{2}} {\text{O}}\left( {\text{l}} \right) + * \to *{\text{O}} + {\text{H}}_{{2}} \left( {\text{g}} \right)} \right] = G_{{*{\text{O}}}} {-}G_{*} {-}\left[ {G_{{{\text{H2O}}({\text{l}})}} {-}G_{{{\text{H2}}({\text{g}})}} } \right]$$

16 $$\Delta G_{{*{\text{OH}}}} = \Delta G\left[ {{\text{H}}_{{2}} {\text{O}}\left( {\text{l}} \right) + * \to *{\text{OH}} + {1}/{\text{2 H}}_{{2}} \left( {\text{g}} \right)} \right] = G_{{*{\text{OH}}}} {-}G_{*} {-}\left[ {G_{{{\text{H2O}}({\text{l}})}} {-}{1}/{2}G_{{{\text{H2}}({\text{g}})}} } \right]$$

17 $$\Delta G_{{*{\text{OOH}}}} = \, \Delta G\left[ {{\text{2H}}_{{2}} {\text{O}}\left( {\text{l}} \right) + * \to *{\text{OOH}} + {3}/{\text{2H}}_{{2}} \left( {\text{g}} \right)} \right] = G_{{*{\text{OOH}}}} {-}G_{*} {-}\left[ {{2}G_{{{\text{H2O}}({\text{l}})}} {-}{3}/{2}G_{{{\text{H2}}}} \left( {\text{g}} \right)} \right]$$

Note: the free energy of oxygen is not a correction in DFT calculation, so the free energy of oxygen is usually calculated from H_2_O and H_2_. The above adsorption energy values of ΔG_*O_, ΔG_*OH_, and ΔG_*OOH_ have been revised.

## Results and Discussion

### Theoretical Design of Co@C-O-Cs Catalyst

**Fig. 1:**
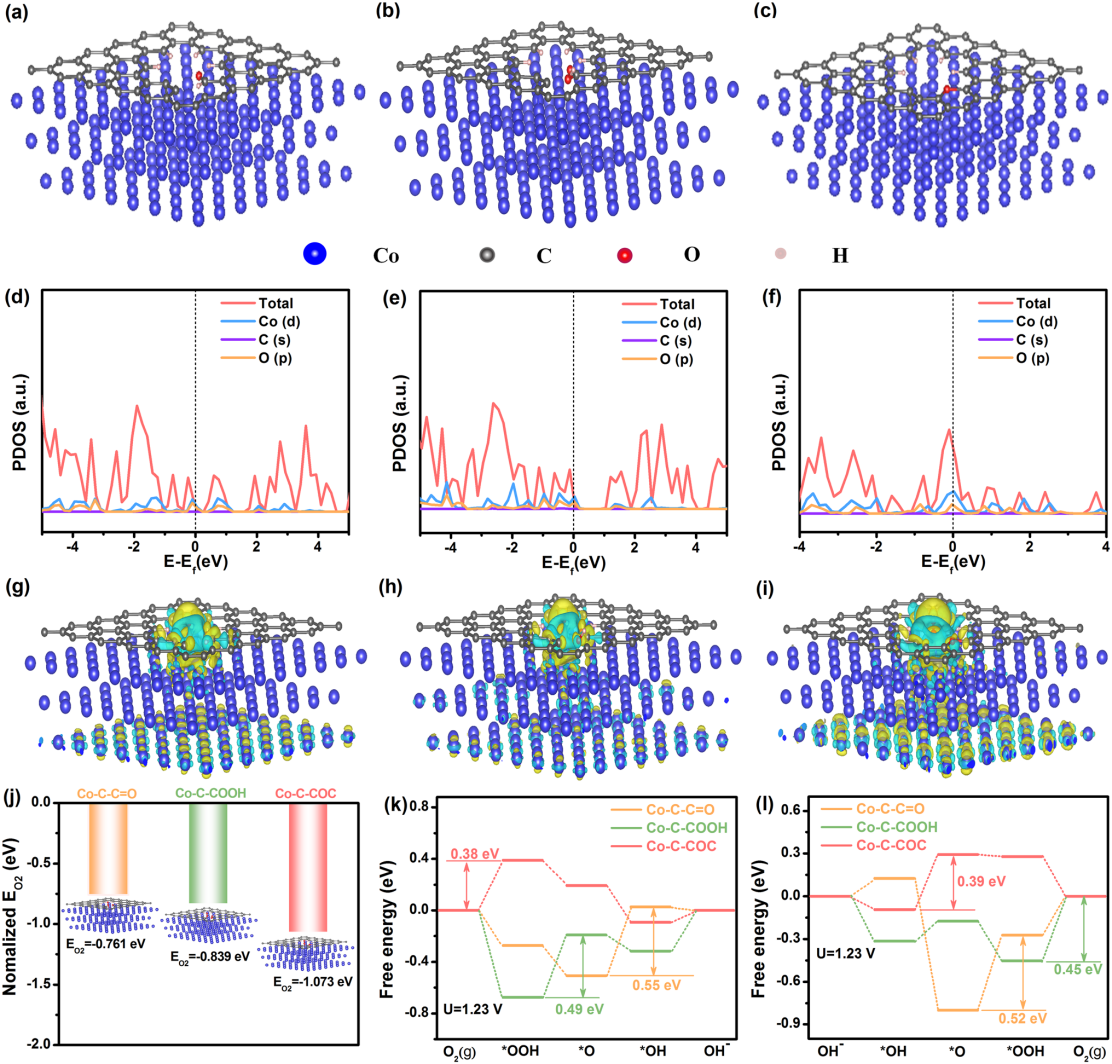
Side view of three theoretical models: **a** Co–C–C = O, **b** Co–C–COOH, and **c** Co–C–COC; Projected total state density of **d** Co–C–C = O, **e** Co–C–COOH, and **f** Co–C–COC; Side view of three theoretical models with a charge density difference of **g** Co–C–C = O, **h** Co–C–COOH, and **i** Co–C–COC, where the yellow and cyan represents electron depletion and accumulation, respectively; **j** O_2_ absorption energy values on Co–C–C = O, Co–C–COOH, and Co–C–COC models; **k** Gibbs free energy plots of ORR at *U* = 1.23 V, and **l** Gibbs free energy plots of OER at *U* = 1.23 V on Co–C–C = O, Co–C–COOH, and Co–C–COC models

The effect of different oxygen-containing active sites in the Co@C–O–Cs catalysts on their bifunctional ORR/OER performance was evaluated by DFT calculations. The Co–C–C = O, Co–C–COOH, and Co–C–COC models were constructed for theoretical analysis (Figs. 1a-c and S1). As shown in Fig. S2, the calculated band structures show that the gap between the conduction and valence band of the three theoretical models are negative, indicating that they have no obvious band gap and have strong electrical conductivity and metallic properties, which will facilitate electron transport. The electronic projected density of states (PDOS) of Co–C–C = O, Co–C–COOH, and Co–C–COC is shown in Fig. 1d–f. Compared with the Co–C–C = O and Co–C–COOH models, the Co–C–COC shows a slight increase in electron-occupied states near the Fermi level, indicating that it has a higher electrical conductivity and carrier density, which contribute to the enhancement of electron transfer capacity between the catalyst surface and the adsorbed intermediates. Combined with the *d*-band theory, the adsorption strength and stability of active sites are highly dependent on the electron filling degree of antibonding orbitals between the atomic orbitals of oxygen-containing intermediates and the *d*-electron state of metal [43, 44]. The obvious increase in the *d*-band center in Co–C–COC indicates that the presence of –C–O–C species may enhance the adsorption capacity of Co–C–COC sites for oxygen intermediates during ORR and OER processes. The charge density difference analysis of three oxygen-containing functional groups was further performed (Figs. 1g–i and S3). Compared with the Co–C–C = O and Co–C–COOH models, the Co–C–COC sites has a higher local charge density, indicating that the electrons are mainly concentrated on carbon atoms. These accumulated electrons can be transferred to oxygen intermediates when O_2_ is adsorbed on the Co–C–COC active sites [45, 46]. The Co–C–COC model has a large overlap between O-2*p* states and Co-3*d* states (Fig. S4), further confirming the presence of abundant –C–O–C active sites can improve the oxygen adsorption capacity of Co@C–O–Cs catalyst, which is consistent with the above differential charge results. Moreover, the O_2_ adsorption energy of the Co–C–COC active sites is −1.073 eV, which is lower than that of the Co–C–C = O active sites of (−0.761 eV) and Co–C–COOH active sites (−0.839 eV) (Fig. 1j).

The ORR/OER pathways on the three kinds of oxygen-containing active sites were further disclosed. Both the ORR and OER processes consist of four adsorbed intermediates: *OO, *OOH, *O, and *OH (* refers to active sites) (Fig. S5) [47]. In all ORR processes, the adsorption free energies of all three models show a decreasing trend at *U* = 0 V (Fig. S6a), indicating that the reaction can proceed spontaneously. At the potential *U* = 1.23 V, the overpotential of Co–C–COC active sites is 0.38 eV (Fig. 1k), which is lower than that of Co–C–C = O active sites (0.55 eV) and Co–C–COOH active sites (0.49 eV). These results indicate that the ORR activity of the Co@C–O–Cs catalyst is dominated by the strong electronic interaction between the metallic Co and oxygen-rich carbon skeleton. For OER, the free energy of these four elementary reactions tends to rise at the potential of *U* = 0 V (Fig. S6b). As shown in Fig. 1l, the Co–C–COC active sites exhibit an overpotential of 0.39 eV, which is smaller than that of Co–C–C = O active sites (0.52 eV) and Co–C–COOH active sites (0.45 eV). This low overpotential can effectively promote the kinetics of the OER process of Co@C–O–Cs catalyst. The lower ORR and OER reaction energy barriers of Co@C–O–Cs catalyst may be mainly attributed to the fast adsorption/desorption of oxygen-containing intermediates on the Co–C–COC active sites [48]. The proposed ORR/OER pathways on the Co@C–O–Cs catalyst is shown in Fig. S7. Therefore, the oxygen-containing active sites can effectively regulate the adsorption of intermediates on the Co@C–O–Cs catalyst surface, thus promoting the intrinsic activity of ORR/OER.

### Construction and Characterization of Co@C-O-Cs Catalyst

**Fig. 2:**
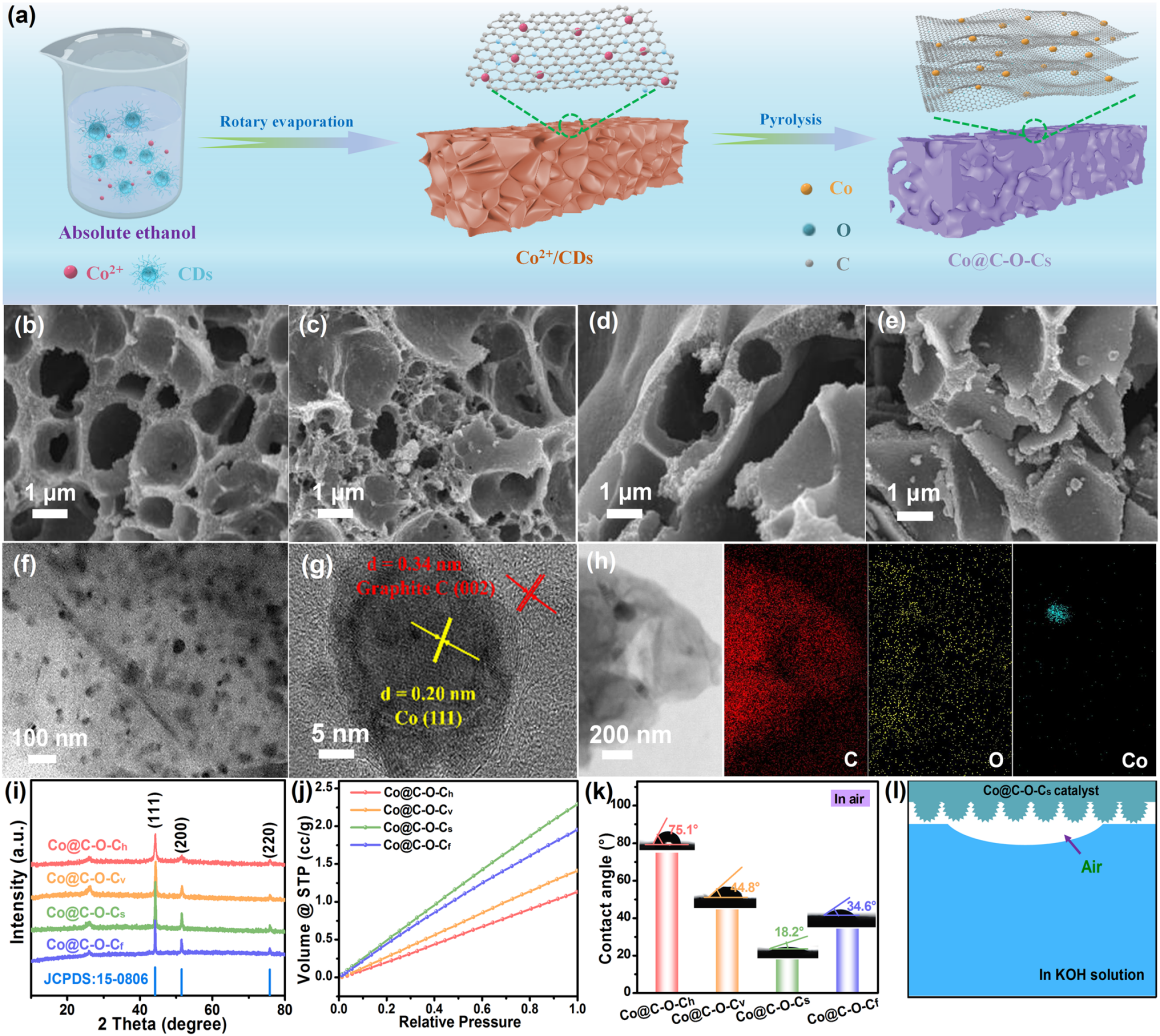
**a** Schematic fabrication procedure of Co@C–O–Cs catalyst; **b**–**e** SEM images of Co@C–O–Ch, Co@C–O–Cv, Co@C–O–Cs, and Co@C–O–Cf; **f–h** TEM, HRTEM, and mapping image of Co@C–O–Cs catalyst; **i** XRD patterns, **j** oxygen adsorption curve, **k** contact angle of the bubble in KOH solution of Co@C–O–Ch, Co@C–O–Cv, Co@C–O–Cs, and Co@C–O–Cf; **l** Schematic aerophilicity of Co@C–O–Cs catalyst in KOH solution

Inspired by DFT calculations, a CDs-assisted synthesis strategy was proposed to construct a three-dimensional sponge-like Co@C–O–Cs catalyst with encapsulated abundant Co NPs and oxygen-rich active sites, as shown in Fig. 2a. Initially, the carbon dots (CDs) with oxygen-rich functional groups were fabricated by condensation reaction between sodium hydroxide and acetaldehyde at 25 °C. Then, CDs and cobalt salt were incorporated into ethanol. Since the used CDs have been proven to be extremely well-dispersed in ethanol, a brownish-yellow solution with a homogeneous dispersion can be obtained after stirring, where partial Co^2+^ may be coordinated with the oxygen-rich functional groups of CDs by the electrostatic interaction. When ethanol in the mixed solution was completely evaporated by rotary evaporation, a uniformly dispersed dark brown aggregate was obtained. Finally, the desired Co@C–O–Cs catalyst with abundant Co NPs and oxygen-containing active sites was obtained by annealing under the Ar atmosphere. During the heat treatment, the oxygen-rich CDs can be decomposed at high temperature to generate a large amount of gas and some carbon atoms evolved into graphitized carbon. Meanwhile, the cobalt ions can be reduced to Co NPs by the carbon. Under the catalysis role of Co NPs and the rapid expansion of gas, the aggregated zero-dimensional CDs can transform into three-dimensional (3D) porous carbon sponge. Co NPs can be highly dispersed within the carbon sponge during the rapid volume expansion process [49].

Scanning electron microscopy (SEM) images of the as-obtained Co@C–O–Ch, Co@C–O–Cv, Co@C–O–Cs, and Co@C–O–Cf samples at 600, 800, 1000, and 1200 °C are shown in Figs. 2b–c and S8. It can be seen that Co@C–O–Cs sample exhibits a 3D carbon sponge structure with large vesicle pores, while Co@C–O–Ch, Co@C–O–Cv, and Co@C–O–Cs samples exhibit honeycomb-like, vesicles-like, and flake-like structure, respectively. With the increase in carbonization temperature, the vesicle pores and fluffy degree of carbon sponge gradually become larger; however, the carbon skeleton is broken, resulting in the decrease in vesicle pores when the temperature reaches 1200 °C. The fluffy 3D oxygen-rich carbon sponge and the interdigitated macropore may facilitate the exposure of active sites and mass transport during the catalysis process [50]. From the transmission electron microscopy images of Figs. 2f and S9(a, d, g), it can be found that metallic Co NPs with an average size of ca. 20 nm are dispersed in the 3D porous carbon sponge, effectively increasing the number of active sites [51]. The dispersed Co NPs could result from the cooperative coordination between Co^2+^ and C–O–C functional groups in CDs, which suppresses the agglomeration of metallic Co NPs during high-temperature carbonization. When the temperature is lower than 800 °C, the bivalent cobalt species cannot be completely reduced to metallic Co. For Co@C–O–C, the high-resolution TEM images in Figs. 2g and S9(b, e, h) show the lattice spacing of 0.20 and 0.34 nm, which corresponds to the (111) plane of Co NPs and the (002) plane of graphite phase, respectively [52]. The selected area electron diffraction pattern of Co@C–O–Cs catalyst (Fig. S10) verifies the (111), (200), and (220) lattice planes of Co NPs, respectively [53, 54]. The lattice of graphite phase indicates that the reduced cobalt NPs may act as a catalyst to boost the formation of fluffy porous carbon sponge and the graphitization of carbon. The element distribution images in Figs. 2h and S9 (c, f, i) show that the Co, O, and C elements are uniformly distributed in the Co@C–O–Cs catalyst.

Based on thermogravimetric analysis under Ar atmosphere (Fig. S11), the precursor mass decreases rapidly when the temperature rises. The large mass loss before 500 °C should be caused by the decomposition of CDs, and then the slight mass loss may result from the reduction of Co^2+^ and graphitization of carbon. The X-ray diffraction patterns in Fig. 2i show that the as-obtained Co@C–O–Ch, Co@C–O–Cv, Co@C–O–Cs, and Co@C–O–Cf have identical diffraction peaks at 44.2°, 51.5°, and 75.8°, which are well-indexed to the (111), (200), and (220) planes of Co (PDF#15–0806) [55, 56]. As the temperature increases from 600 to 1200 °C, the diffraction peaks of metallic Co become sharper, indicating an increase in crystallinity. The wide diffraction peak at 25° results from the (002) plane of graphitic carbon, indicating the formation of graphitic structure during the annealing process. According to the TG results of Co@C–O–Ch, Co@C–O–Cv, Co@C–O–Cs, and Co@C–O–Cf in air atmosphere (Fig. S12a), the determined Co contents of four samples are 20.5%, 17.0%, 20.0%, and 30.2%, respectively. X-ray diffraction pattern confirms that the residual oxidation product at 800 °C was Co_3_O_4_ (Fig. S12b). The Raman peaks located at 1340 and 1580 cm^−1^ belong to the D-band (structural defects) and the G-band (graphite structure), respectively [57, 58]. The integrated intensity ratio of the D to G peak (*I*_D_/*I*_G_), which is an indicator of the degree of disorder, was calculated from the Raman spectra of all samples. The results verify that the *I*_D_/*I*_G_ value of the Co@C–O–Cs is 0.84, which is lower than that of Co@C–O–Ch (0.99), Co@C–O–Cv (0.98), and Co@C–O–Cf (0.85), indicates that the graphitization degree of the catalysts increases as the carbonization temperature increases (Fig. S13). As shown in Fig. S14a, all of the isotherms belong to type-IV curve combined with the H4-type hysteresis loop, suggesting the existence of microporous and mesoporous in the Co@C–O–Ch, Co@C–O–Cv, Co@C–O–Cs, and Co@C–O–Cf [59]. The Brunauer–Emmett–Teller (BET) specific surface area of Co@C–O–Cs (203.7 m^2^ g^−1^) is much larger than Co@C–O–Ch (127.3 m^2^ g^−1^), Co@C–O–Cv (140.7 m^2^ g^−1^), and Co@C–O–Cf (155.9 m^2^ g^−1^), indicating that an increase in temperature can induce a larger specific surface area. When the temperature increases to 1200 °C, the carbon sponge structure collapses and the macropores are broken, resulting in the decrease in specific surface area. Moreover, it can be found from Fig. S14b that these samples possess a similar non-uniformly distributed nano-meso-micro-hierarchical porous structure. The hierarchical porous structure of Co@C–O–Cs may facilitate the mass diffusion of oxygen species at the three-phase interface during the catalytic process [60]. The ORR and OER involve a gas–liquid–solid tri-phase interface, the mass transport between the reactant and active sites is a vital factor to determine reaction rate. Therefore, oxygen adsorption isotherms were measured to evaluate the oxygen adsorption property of Co@C-O–Ch, Co@C–O–Cv, Co@C–O–Cs, and Co@C–O–Cf. As shown in Fig. 2j, with the increase in carbonization temperature, the oxygen adsorption capacity becomes larger. However, when the temperature reaches 1200 °C, the adsorption of oxygen decreases due to the collapse of the carbon sponge structure for Co@C–O–Cf. In Fig. S15, the maximum specific surface area of Co@C–O–Cs catalyst for oxygen adsorption can reach up to 2.3 m^3^ g^−1^, which is larger than Co@C–O–Ch (1.1 m^3^ g^−1^), Co@C–O–Cv (1.4 m^3^ g^−1^), and Co@C–O–Cf (1.9 m^3^ g^−1^), indicating the higher adsorption capacity of oxygen for Co@C–O–Cs catalyst [61, 62].

The wettability in air and the adhesion behavior of oxygen bubbles in KOH solution on the as-fabricated catalysts were investigated by contact angle measurement. The contact angle value of KOH solution on the surface of Co@C–O–Cs catalyst in air is 18.2°, which is lower than that of Co@C–O–Ch (75.1°), Co@C–O–Cv (44.8°), and Co@C–O–Cf (34.6°) (Fig. S16). The good wettability and hydrophilicity of the oxygen-rich carbon sponge facilitate the contact between the active sites and electrolyte, thereby potentially improving the ORR/OER catalytic kinetics on the Co@C–O–Cs catalyst [63]. Meanwhile, as shown in Fig. 2k, the contact angle of oxygen bubbles in KOH solution on Co@C–O–Cs catalyst is 45.9°, which is smaller than that of Co@C–O–Ch (90.1°), Co@C–O–Cv (65.9°), and Co@C–O–Cf (60.5°), which strongly demonstrates that the Co@C–O–Cs catalyst has strong adhesion ability of oxygen bubbles, thus showing excellent aerophilicity of the Co@C–O–Cs catalyst surface. Benefiting from the lower surface roughness and loose sponge structure, the Co@C–O–Cs exhibits the high hydrophilicity in air and aerophilicity in KOH solution (Figs. 2l and S17). Based on the Cassie-Baxter wetting regime [64], the loose sponge structure of Co@C–O–Cs improves the adhesion to oxygen bubbles. This strong surface adhesion role can continuously accelerate the diffusion and transfer of O_2_, thus the large amount of reactants at the three-phase interface can boost the ORR/OER kinetics in the alkaline electrolyte [65].

**Fig. 3:**
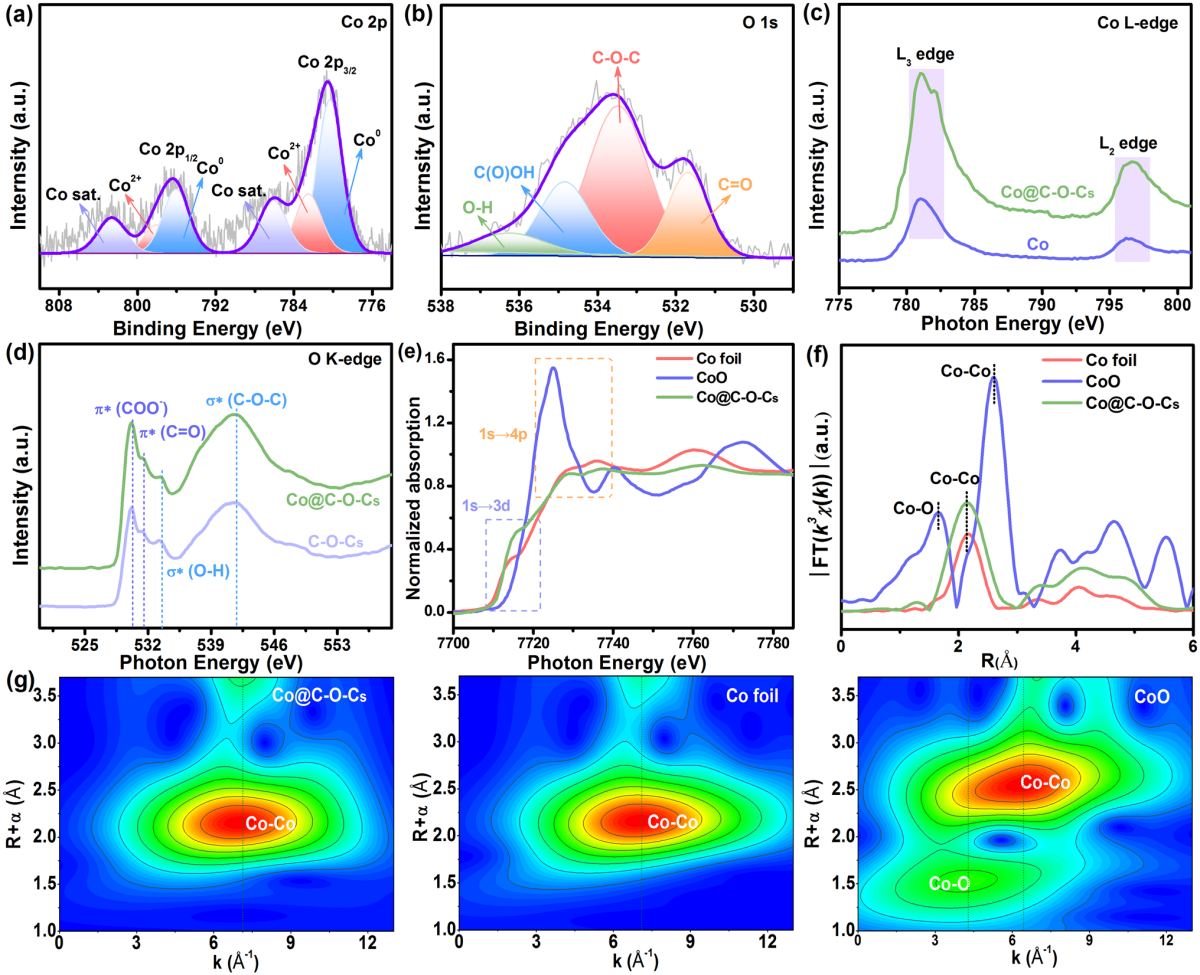
**a** Co 2*p* and **b** O 1*s* high-resolution XPS spectra of Co@C–O–Cs; **c** Co L-edge and **d** O K-edge XANES spectra of Co@C–O–Cs and reference samples; **e** Co K-edge XANES spectra of Co@C–O–Cs and reference samples; **f** Fourier transform spectra of Co@C–O–Cs and reference samples; **g** WT-EXAFS spectra of Co@C–O–Cs and reference samples

The near-surface chemical structure and components of the Co@C–O–C at different temperatures were characterized by X-ray photoelectron spectroscopy (XPS). The XPS full spectrum shows the presence of Co, C, and O elements in all samples (Fig. S18a–d). The high-resolution C 1*s* for Co@C–O–C at different temperatures (Fig. S18e–h) can be divided into four peaks, including C–C (284.7 V), C–O (285.9 V), C = O (286.7 V), and O–C = O (287.7 V) bands [66], demonstrating the presence of oxygen species on the carbon skeleton. As shown in Figs. 3a and S18i-k, two pairs of 2*p*_3/2_/2*p*_1/2_ doublets in the high-resolution Co 2*p* spectrum were deconvoluted into three major peaks, including Co^0^ (779.5 and 795.7 eV), Co^2+^ (781.8 and 797.4 eV), and shakeup satellite (786.6 and 804.9 eV) [67]. Notably, the peak corresponding to Co^0^ is smaller than that of Co^2+^ because small Co NPs are prone to surface oxidation, resulting in the presence of Co–O species on the catalyst surface. This phenomenon can be widely observed in related transition metal catalysts. The high-resolution O 1*s* spectrum (Figs. 3b and S18l-n) can be fitted with C–O–C (533.5 eV), C(O) –OH (535.1 eV), and C = O (532.1 eV) bands [68], further suggesting the formation of abundant oxygen-bearing components in the 3D porous carbon sponge.

The soft/hard X-ray absorption spectroscopy analysis was performed to identify the local electronic structure and coordination environment of Co, C, and O atoms in the Co@C–O–Cs catalyst. The C K-edge near-edge X-ray absorption fine structure (NEXAFS) spectra of Co@C–O–Cs (Fig. S19) displays two typical spectroscopic peaks at 285.8 eV (C = C π*) and 292.3 eV (C–C σ*), implying a typical *sp*^2^ hybrid carbon structure [69]. Moreover, the peak at 288.9 eV is a fingerprint of the *sp*^3^ carbon, evidencing the existence of C–O–C in the carbon lattice [70]. The Co L-edge NEXAFS spectra of Co@C–O–Cs (Fig. 3c) show two prominent peaks, including Co L_3_ at 781.1 eV and Co L_2_ at 796.4 eV, indicating the 2*p* core–hole spin–orbit interaction between Co and carbon skeleton [71]. The O K-edge NEXAFS spectra of Co@C–O–Cs in Fig. 3d exhibit three characteristic peaks, including π*(COO^−^) (530.2 eV), π*(C = O) (531.6 eV), and σ*(C–O–C) (541.7 eV), respectively [72, 73]. The Co K-edge X-ray absorption near edge structure spectrum of Co@C–O–Cs shows a pre-edge feature at 7708 eV, which is attributed to the 1*s* → 3*d* electron transition (Fig. 3e). The peak intensity of Co@C–O–Cs (1*s* → 4*pz*) is between that of Co foil and CoO reference samples, confirming the existence of interfacial interaction between the Co NPs and porous carbon sponge. Moreover, the near-edge absorption threshold of Co K-edge lies between those of Co foil (Co^0^) and CoO (Co^2+^), demonstrating the average valence state of Co is between 0 and + 2 mixed oxidation states [74]. The Fourier transform EXAFS curve of Co@C–O–Cs in Fig. 3f presents a peak at 2.1 Å, which is attributed to the Co–Co scattering paths [75]. Because of the high resolution of wavelet-transforming (WT)-EXAFS analysis, it has become an effective method to discriminate the coordination environment. Compared to the Co foil and CoO samples, the WT-EXAFS plot of Co@C–O–Cs shows two sole contour peak intensity maximums at around 7.1 Å^−1^ (Fig. 3g), which corresponds to the Co–Co coordination [76]. This phenomenon indicates the presence of Co–C–O–C coordination configuration in the Co@C–O–Cs catalyst.

### Electrochemical Evaluation

**Fig. 4:**
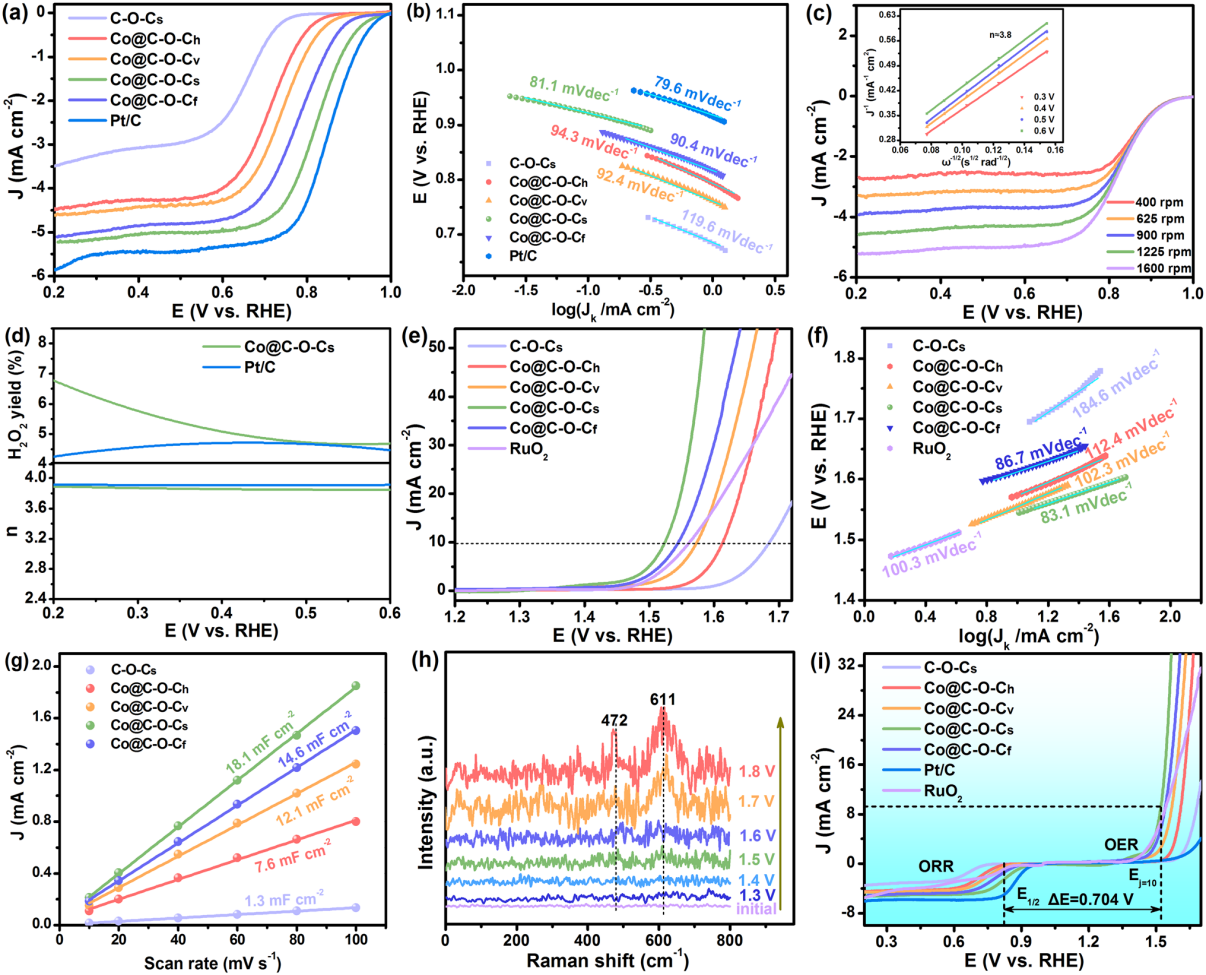
**a** ORR LSV measurements in O_2_-saturated 1.0 mol L^−1^ KOH solution at 1600 rpm; **b** Tafel plots of ORR process; **c** LSV curves of Co@C–O–Cs catalyst under different rotation speeds (inset: K-L plots of the Co@C–O–Cs catalyst); **d** Transferred electron number and H_2_O_2_% of Co@C–O–Cs and Pt/C catalysts; **e** OER LSV measurements in O_2_-saturated 1.0 mol L^−1^ KOH solution; **f** Tafel plots of OER process; **g** *C*_dl_ curves of various catalysts; **h** In situ Raman spectra of Co@C–O–Cs catalyst in 1.0 mol L^−1^ KOH solution at different potentials for OER process; **i** Overall polarization curves of various catalysts

The catalytic activities of Co@C–O–Cs catalysts and benchmarked commercial Pt/C toward ORR were evaluated by a typical three-electrode system in an O_2_-saturated 1.0 mol L^−1^ KOH solution. Cyclic voltammetry profile of Co@C–O–Cs displays an obvious cathodic ORR peak at 0.77 V in the O_2_-saturated electrolyte, compared with that in the N_2_-saturated electrolyte (Fig. S20), implying effective catalytic activity for oxygen reduction. Moreover, linear sweep voltammetry (LSV) was conducted to disclose their kinetics and activities for ORR. The Co@C–O–Cs catalyst affords a remarkable ORR performance with a half-wave potential (*E*_1/2_) of 0.82 V and an onset and half-wave potential (*E*_onset_) of 0.90 V (Figs. 4a and S21), which are comparable to the benchmarked Pt/C (*E*_1/2_ = 0.86 V and *E*_onset_ = 0.93 V), and obviously superior than those of C–O–Cs (*E*_1/2_ = 0.64 V and *E*_onset_ = 0.72 V), Co@C–O–Ch (*E*_1/2_ = 0.71 V and *E*_onset_ = 0.79 V), Co@C–O–Cv (*E*_1/2_ = 0.73 V and *E*_onset_ = 0.83 V), Co@C–O–Cf (*E*_1/2_ = 0.75 V and *E*_onset_ = 0.85 V), and the most of recently reported catalysts in Table S1. The corresponding Tafel plots of Co@C–O–Cs catalyst under alkaline conditions is 81.1 mV dec^−1^ (Fig. 4b), which is lower than those of C–O–Cs (119.6 mV dec^−1^), Co@C–O–Ch (94.3 mV dec^−1^), Co@C–O–Cv (92.4 mV dec^−1^), Co@C–O–Cf (90.4 mV dec^−1^), and is comparable to that of the commercial Pt/C catalyst (79.6 mV dec^−1^), suggesting the faster ORR kinetics on Co@C–O–Cs catalyst. To investigate the ORR mechanism, the electron transfer number (*n*) was further determined by analyzing LSV curves at different rotating speeds using the Koutecky–Levich (K–L) equations (Figs. 4c and S22). The K–L plots have shown good linearity relationships (inset of Fig. 4c), confirming the first-order kinetics process of the Co@C–O–Cs catalyst, which is dependent on the concentration of dissolved oxygen in the alkaline medium [77]. Moreover, the electron transfer number of Co@C–O–Cs is calculated as 3.8, which is close to 4.0 of benchmarked Pt/C, suggesting a four electron transfer pathway during the ORR process. The ORR pathway was further investigated by the rotating ring-disk electrode (RRDE) measurements (Figs. S23 and 4d). The average H_2_O_2_ yield is 5.6% and the calculated n value is 3.82, which is in agreement with the results obtained from the K–L plot. Therefore, these results verify that the Co@C–O–Cs catalyst exhibits an efficient ORR kinetic process with four-electron transfer reaction mechanism. The stability of Co@C–O–Cs and Pt/C catalysts was assessed by chronoamperometric (CA) measurement within an O_2_-saturated 1.0 mol L^−1^ KOH solution for 12,000 s. The current density of Co@C–O–Cs is maintained at 93.8% (Fig. S24a), which is higher than the 78.9% retention rate of Pt/C, demonstrating the high catalytic stability of the Co@C–O–Cs catalyst. In addition, a slight decay in the *E*_1/2_ of Co@C–O–Cs (12.5 mV decay in *E*_1/2_) after 1000 cycles by comparing with that of Pt/C catalyst (18.6 mV decay in *E*_1/2_) (Fig. S24b), indicating excellent ORR durability of the Co@C–O–Cs catalyst because the Co NPs are encapsulated in the porous oxygen-rich carbon sponges.

The OER catalytic performance of Co@C–O–C was further evaluated by LSV in 1.0 mol L^−1^ KOH solution. The Co@C–O–Cs presents a low overpotential value of 294 mV at a current density of 10 mA cm^−2^ (Fig. 4e), which is obviously lower than C–O–Cs (450 mV), Co@C–O–Ch (380 mV), Co@C–O–Cv (340 mV), Co@C–O–Cf (320 mV), commercial RuO_2_ catalyst (330 mV), and those of previously reported catalysts in Table S2. Moreover, the Tafel slope of Co@C–O–Cs is 83.1 mV dec^−1^, which is much lower than those of C–O–Cs (184.6 mV dec^−1^), Co@C–O–Ch (112.4 mV dec^−1^), Co@C–O–Cv (102.3 mV dec^−1^), Co@C–O–Cf (86.7 mV dec^−1^), and comparable to that of RuO_2_ (100.3 mV dec^−1^) (Fig. 4f), indicating faster OER kinetics on Co@C–O–Cs catalyst [78]. From the Nyquist plots and impedance fitting data shown in Fig. S25 and Table S3, the Co@C–O–Cs catalyst exhibits the smallest charge-transfer resistance (*R*_ct_ = 15.15 Ω) and the highest constant phase elements (CPE = 9.85 × 10^–4^ mF), reflecting that more efficient electron transfer occurs at the Co@C–O–Cs/electrolyte interface during the catalytic reaction. After a 12,000 continuous reaction, the Co@C–O–Cs catalyst retains 93.7% of the initial current, which is much lower than the commercial RuO_2_ catalyst (86.5%) (Fig. S26a). Additionally, a negligible activity loss of Co@C–O–Cs can be observed after 1000 potential cycles (∼7.2 mV at *E*_j=10_), which is lower than that of commercial RuO_2_ (19.4 mV) (Fig. S26b). These results suggest that the Co@C–O–Cs catalyst exhibits excellent OER electrochemical stability in alkaline solution. The electrochemically active surface area (ECSA) was obtained to demonstrate the origin of the intrinsic activity of the Co@C–O–Cs catalyst based on the electrochemical double layer capacitance (*C*_*dl*_) at different scan rates (Fig. S27). The calculated *C*_*dl*_ of Co@C–O–Cs is 18.1 mF cm^−2^ (Fig. 4g), which is significantly higher than those of C–O–Cs (1.3 mF cm^−2^), Co@C–O–Ch (7.6 mF cm^−2^), Co@C–O–Cv (12.1 mF cm^−2^), and Co@C–O–Cf (14.6 mF cm^−2^), resulting in the excellent mass transportation for the Co@C–O–Cs catalyst. To demonstrate the stability of the Co@C–O–Cs catalyst, the morphology, composition, and elemental electronic states of samples after OER stability tests were characterized, as shown in Fig. S28. It can be seen from Fig. S28a-c that the Co NPs are still distributed in the carbon sponge after cycling and no corrosion occurs. Furthermore, the XRD pattern of the Co@C–O–Cs catalyst after cycling shows the same characteristic peaks with before cycling (Fig. S28d), indicating that no phase transition occurs during cycling. The XPS results of Co@C–O–Cs catalyst (Fig. S28e–g) also show that the positions of the Co, C, and O peaks hardly change. These results suggest that the Co@C–O–Cs catalyst still remains its initial structure after OER stability test.

The possible catalytic mechanism and surface structure changes of Co@C–O–Cs catalyst during OER under controlled overpotentials in 1.0 mol L^−1^ KOH solution were measured by in situ Raman spectroscopy technique (Fig. S29). As shown in Fig. 4h, there is no feature peak of lattice vibration between 400 and 800 cm^−1^ under the open-circuit condition. Moreover, no new peak can be observed when the voltage is lower than 1.5 V, indicating that OER does not proceed. When the potential rises from 1.5 to 1.8 V, a newly formed vibrational peak at 472 cm^−1^ indicates the formation of *β*-Co(OH)_2_ species [79]. Meanwhile, a more intense peak at 611 cm^−1^ may be attributed to the formation of CoOOH intermediate under the harsh oxidative environment [80, 81]. The increased peak intensity of the CoOOH intermediate is larger than that of *β*-Co(OH)_2_ due to the transformation of *β*-Co(OH)_2_ into CoOOH intermediate. The formation of these intermediates over 1.5 V may result in the low OER overpotential of Co@C–O–Cs catalyst. These results demonstrate that the partial Co–C-COC active sites may be converted into CoOOH species under the high potential and strong alkali environments, which have the potential to boost OER and resist the destruction of Co@C–O–Cs catalyst. The potential gap (Δ*E*) between the OER overpotential at 10 mA cm^−2^ and ORR half-wave potential (Δ*E* = *E*_j=10_ − *E*_1/2_) is generally used to evaluate the bifunctional activity and reversible electrochemical properties [82]. As shown in Figs. 4i and S30, the Co@C–O–Cs catalyst has a comparable Δ*E* value of 0.704 V, which is markedly lower than that of C–O–Cs (1.04 V), Co@C–O–Ch (0.90 V), Co@C–O–Cv (0.84 V), Co@C–O–Cf (0.80 V), Pt/C (1.03 V), commercial RuO_2_ (0.98 V), and some bifunctional ORR/OER catalysts previously reported in Table S4. Therefore, the Co@C–O–Cs has great potential as a bifunctional catalyst as air cathode for rechargeable ZABs.

### Rechargeable ZABs

**Fig. 5:**
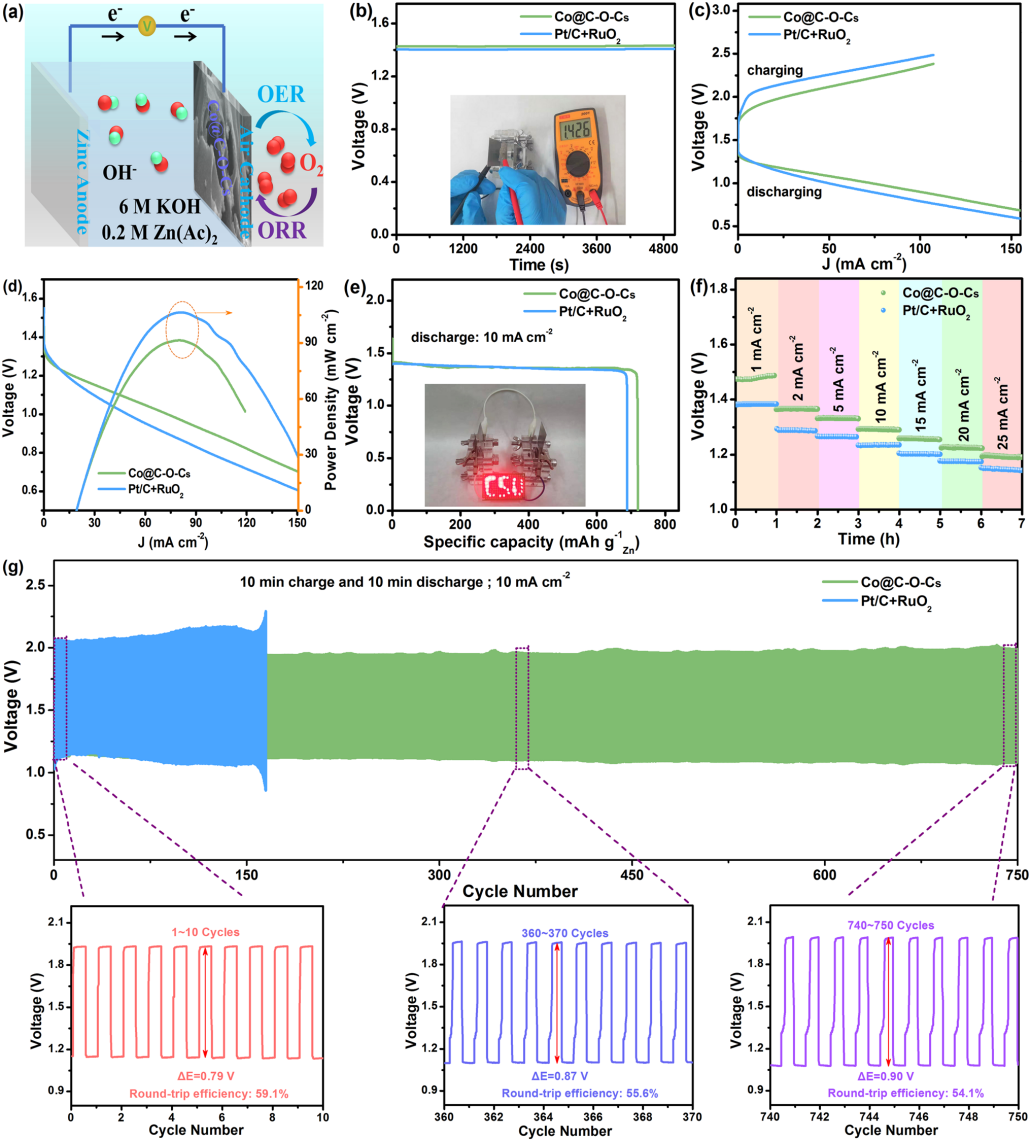
**a** Schematic diagram of rechargeable liquid ZAB with Co@C–O–Cs catalyst; **b** Open-circuit plots, **c** charge and discharge polarization curves, **d** discharge polarization curves and the corresponding power density curves, and **e** discharge polarization curves at the current density of 10 mA cm^−2^ of rechargeable liquid ZABs with Co@C–O–Cs and Pt/C + RuO_2_ catalysts (inset: photograph of a ‘CSU’ pattern formed by LEDs driven by two rechargeable liquid ZABs with Co@C–O–Cs catalyst); **f** Discharge curves at various current densities; **g** Discharge–charge cycling performance of the rechargeable liquid ZABs with Co@C–O–Cs and Pt/C + RuO_2_ catalysts at 10 mA cm^−2^

Considering the excellent bifunctional activity of Co@C–O–Cs catalyst, a home-made rechargeable liquid ZAB was assembled. As shown in Figs. 5a and S31, the liquid ZAB consists of a polished Zn plate as anode, Co@C–O–Cs catalyst as cathode, 6.0 mol L^−1^ KOH solution with 0.2 mol L^−1^ zinc acetate (Zn(Ac)_2_) as electrolyte. The open-circuit voltage of Co@C–O–Cs-based ZAB is 1.426 V, which is closed to Pt/C + RuO_2_-based ZAB (1.40 V) (Fig. 5b). The discharge and charge curves in Fig. 5c show that under different current densities, the Co@C–O–Cs-based liquid ZAB has the smaller voltage gaps than the control device based on Pt/C + RuO_2_. As shown in Fig. 5d, the Co@C–O–Cs-based liquid ZAB achieves a current density of 73.1 mA cm^−2^ at 1.0 V and a peak power density of 106.4 mW cm^−2^ at 164.5 mA cm^−2^, which are superior to those of Pt/C + RuO_2_-based ZAB (49.4 mA cm^−2^ at 1.0 V, maximum 88.7 mW cm^−2^). Additionally, the specific capacity of the Co@C–O–Cs-based liquid ZAB can reach 720.7 mAh g^−1^, which is comparable to those of the Pt/C + RuO_2_-based ZAB (690.7 mAh g^−1^; Fig. 5e). A light-emitting diode (LED) with ‘CSU’ (Central South University) screen can be powered by two liquid ZABs in series with the Co@C–O–Cs catalyst as the air cathode (inset of Fig. 5e), demonstrating its potential application in energy conversion devices. As increasing current density, the discharge potential plateau decreases and remains stable. Even at 25 mA cm^−2^, the Co@C–O–Cs-based liquid ZAB still exhibits a high discharging potential of 1.19 V (Fig. 5f), suggesting its excellent high-rate performance. Furthermore, the cycling stabilities for Co@C–O–Cs and Pt/C + RuO_2_-based rechargeable liquid ZABs was investigated under conditions of charge/discharge at 10 mA cm^−2^ (Fig. 5g). The Co@C–O–Cs-based liquid ZAB exhibits an ultra-stable operation window of over 750 cycles, which is much better than Pt/C + RuO_2_-based ZAB (300 cycles). Interestingly, the Co@C–O–Cs-based ZAB exhibits a small charge–discharge voltage gap of 0.79 V at 1.14 V discharge voltage and 1.93 V charge voltage, and the high round-trip efficiency is 59.1%. After 360 cycles, the charge/discharge voltage gap only varies to 0.87 V and round-trip efficiency only decreases to 55.6%. Even after 750 cycles, it still maintains good stability. Therefore, the Co@C–O–Cs-based rechargeable liquid ZAB exhibits the better stability performance than that of Pt/C + RuO_2_ and some recently reported batteries based on non-precious metal catalysts in Table S5.

**Fig. 6:**
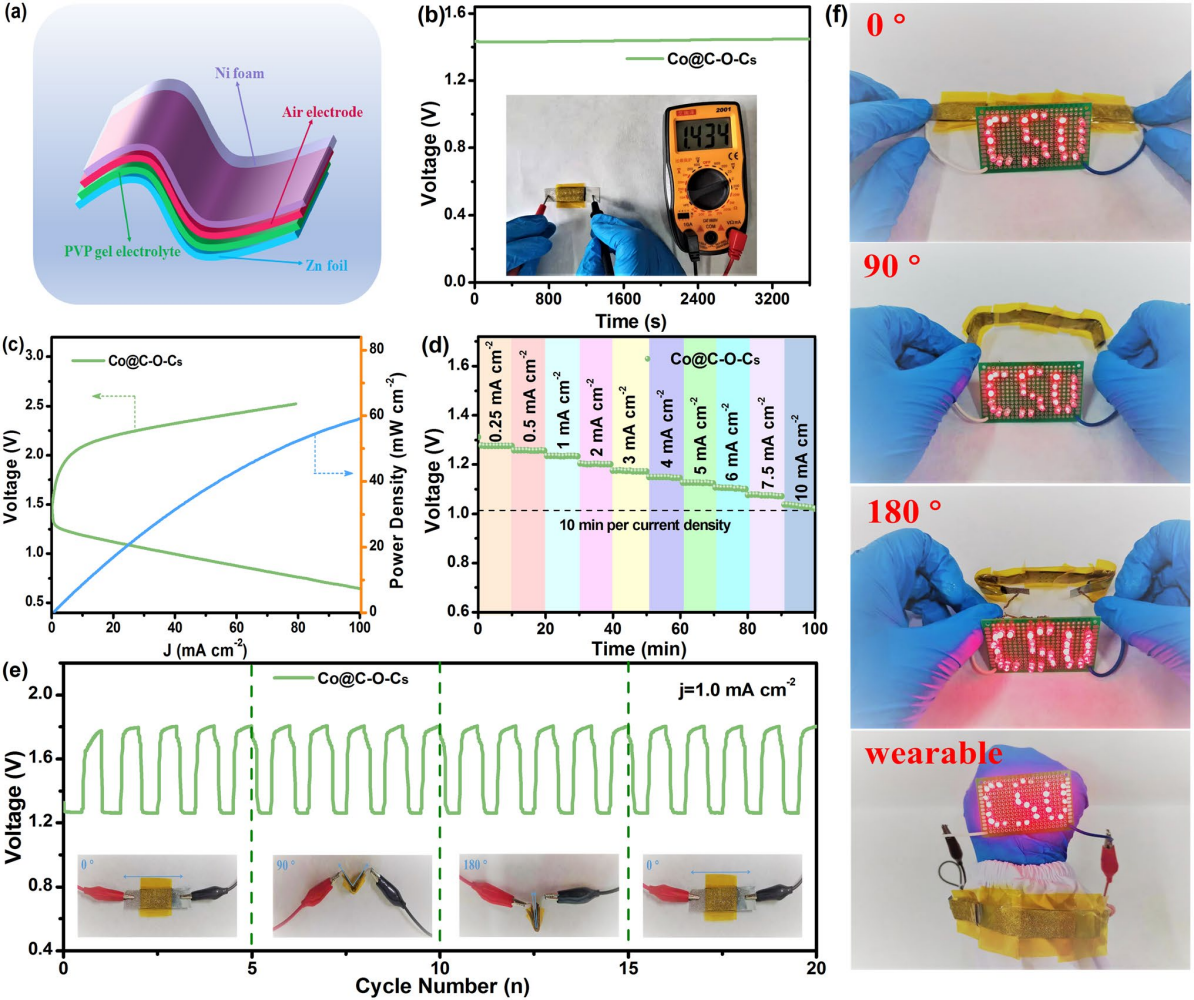
**a** Schematic diagram of rechargeable flexible all-solid-state ZAB with Co@C–O–Cs catalyst; **b** Open-circuit plots and **c** charge and discharge polarization curves and the corresponding power density curves of rechargeable flexible all-solid-state with Co@C–O–Cs catalyst; **d** Discharge curves of the battery at various current densities; **e** Cycling stability of rechargeable flexible all-solid-state ZAB with different bending angles; **f** Photographs of LED lightened by three flexible ZABs connected in series when bent at various angles and the three-series all-solid-state ZABs as a wearable bracelet to light up the LED

With the increasing demand for flexible and portable electronic devices, all-solid-state ZABs are emerging as a promising alternative for efficient and sustainable power supply. Flexible all-solid-state ZABs were further fabricated with Co@C–O–Cs supported on nickel foam as air cathode, alkaline polyvinyl alcohol (PVA) gel as electrolyte, Zn foil as metal anode (Fig. 6a). The Co@C–O–Cs-based all-solid-state ZAB exhibits an open-circuit voltage of 1.43 V (Fig. 6b) and a high power density of 59.1 mW cm^−2^ (Fig. 6c), which is comparable to those of recently reported bifunctional catalysts in Table S6. As shown in Fig. 6d, the discharge voltage gradually decreases from 1.28 to 1.03 V with the current density varying from 1 to 12 mA cm^−2^. Moreover, as shown in Fig. 6e, the flexible Co@C–O–Cs-based ZAB can charge and discharge for a long time at a constant current density of 1.0 mA cm^−2^, even if the battery is bent at different angles (including 90° and 180°). Under the initial state (0°), the flexible all-solid-state battery exhibits an ultra-low voltage polarization of 0.50 V (charge and discharge voltages are 1.77 and 1.27 V, respectively), and the round-trip efficiency is 71.5%. A low-voltage polarization and high round-trip efficiency can be still remained at 90° (0.55 V, 69.4%) and 180° (0.56 V, 69.1%). Moreover, the voltage polarization rate and round-trip efficiency can maintain at 0.58 V and 68.5% after the battery was returned to the initial state, indicating the excellent flexibility of the Co@C–O–Cs-based all-solid-state ZAB. A working prototype that contains three series of flexible all-solid-state ZABs based on the Co@C–O–Cs catalyst was also established. As shown in Fig. 6f, it can successfully light up the LED with ‘CSU’ indicator even bending at different angles. Moreover, the three series of ZABs can be used as wearable bracelets to light up the LED with stable operating conditions. Compared with rechargeable liquid ZABs, this rechargeable all-solid-state ZAB exhibits more attractive features due to their small size, flexibility, high power density, and high safety.

## Conclusions

In summary, the oxygen-respirable Co@C–O–Cs catalyst with oxygen-rich active sites were successfully constructed with CDs-assisted synthesis strategy as the efficient bifunctional ORR/OER catalysts. The hydrophilicity and aerophilicity of Co@C–O–Cs are beneficial to oxygen diffusion and mass transfer. The density functional theory calculations and experimental studies revealed that the Co–O–COC active sites can modulate the local charge density, lower the reaction energy barrier, and enhance the ORR/OER activity. In situ Raman spectroscopy revealed that partial Co–O-COC active site could transform to the Co–OOH intermediate during OER process to maintain high catalysis activity and stability. The Co@C–O–Cs catalyst delivers a half-wave potential of 0.82 V for ORR and a low overpotential of 294 mV at 10 mA cm^−2^ for OER. The Co@C–O–Cs-based ZAB displays a high discharge peak power density of 106.4 mW cm^−2^, high specific capacity of 720.7 mAh g^−1^, and outstanding cycling stability over 750 cycles, which outperforms the Pt/C + RuO_2_. Furthermore, the flexible Co@C–O–Cs-based all-solid-state ZAB exhibits high power density and good flexibility under 0–180° bending degrees. These findings provide new insights into the design of efficient bifunctional catalysts for versatile energy storage and conversion devices.

### Supplementary Information

Below is the link to the electronic supplementary material.

Supplementary file1 (PDF 2642 kb)
