# Supplementary material for: Aerophilic Triphase Interface Tuned by Carbon Dots Driving Durable and Flexible Rechargeable Zn-Air Batteries
Source: Nanomicro Lett. 2023 Jan 3;15:28. doi: 10.1007/s40820-022-00994-3 (PMC9810778; doi:10.1007/s40820-022-00994-3)
Supplement: Supplementary file 1 — Supplementary file1 (PDF 2642 kb) [file 40820_2022_994_MOESM1_ESM.pdf]

Supporting Information for

Aerophilic Triphase Interface Tuned by Carbon Dots Driving Durable and Flexible Rechargeable Zn-Air Batteries

Kuixing Ding[1,#], Yu Ye[1,#], Jiugang Hu[1,*], Liming Zhao[2], Wei Jin[3], Jia Luo[1], Shan Cai[1], Baicheng Weng[1], Guoqiang Zou[1], Hongshuai Hou[1,*], Xiaobo Ji[1]

[1] College of Chemistry and Chemical Engineering, Central South University, Changsha, 410083, P. R. China

[2] College of Standardization, China Jiliang University, Hangzhou 310018, P. R. China

[3] School of Environmental Science and Engineering, Suzhou University of Science and Technology, Suzhou, 215009, P. R. China

#Kuixing Ding and Yu Ye contributed equally to this work.

*Corresponding authors. E-mail: hujiugang@csu.edu.cn (Jiugang Hu); hs-hou@csu.edu.cn (Hongshuai Hou)

Supplementary Figures and Tables

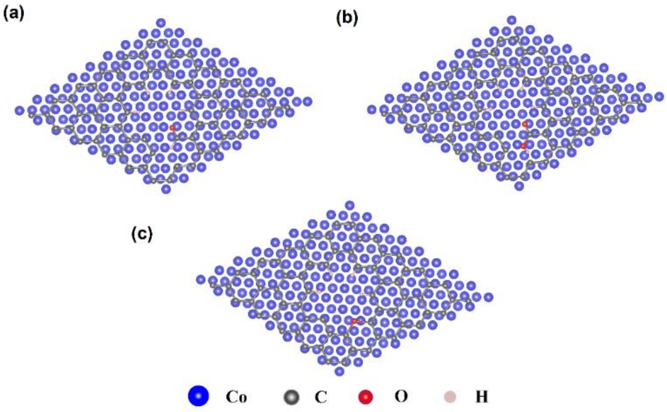

Fig. S1 Top view of the theoretical models: (**a**) Co-C-C=O; (**b**) Co-C-COOH; (**c**) Co-C-COC

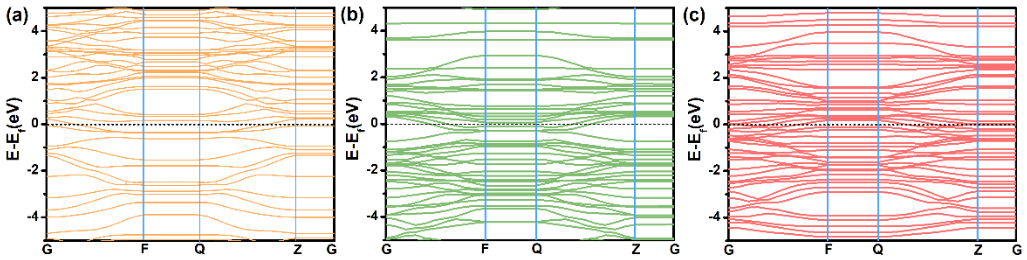

Fig. S2 Calculated band structures of (**a**) Co-C-C=O; (**b**) Co-C-COOH; (**c**) Co-C-COC

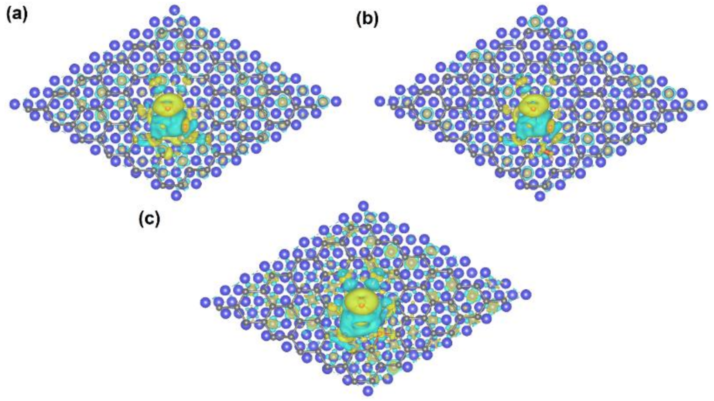

Fig. S3 The top view of the theoretical models with charge density difference of (**a**) Co-C-C=O, (**b**) Co-C-COOH, (**c**) Co-C-COC, the yellow and cyan represent electron depletion and accumulation, respectively

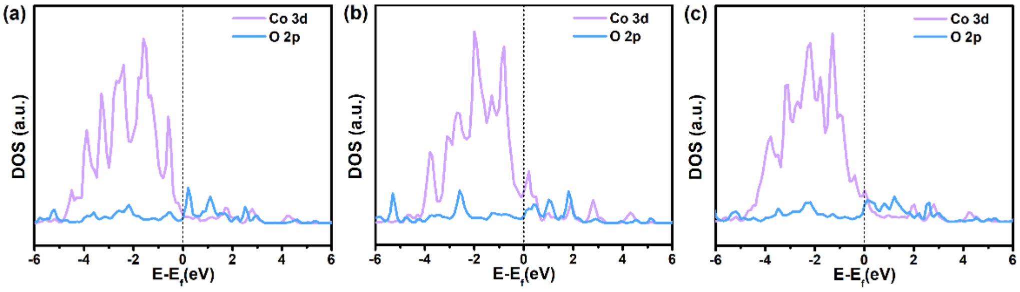

Fig. S4 DOS plots of O atoms on (**a**) Co-C-C=O, (**b**) Co-C-COOH, and (**c**) Co-C-COC models

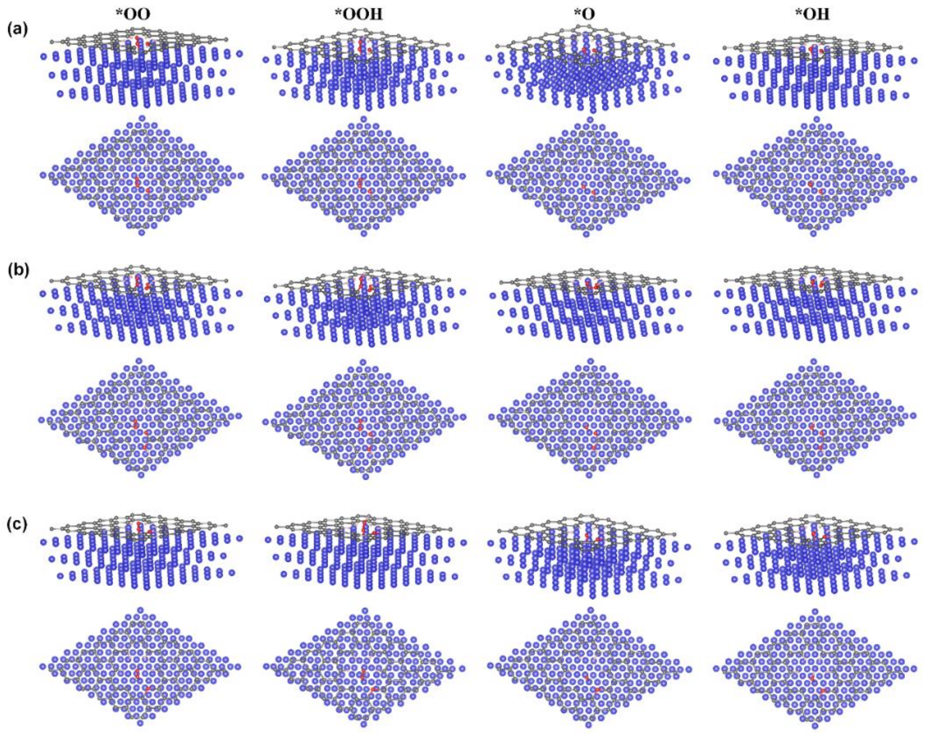

Fig. S5 The optimized geometrical structures of oxygen-containing intermediates on (**a**) Co-C-C=O; (**b**) Co-C-COOH; (**c**) Co-C-COC

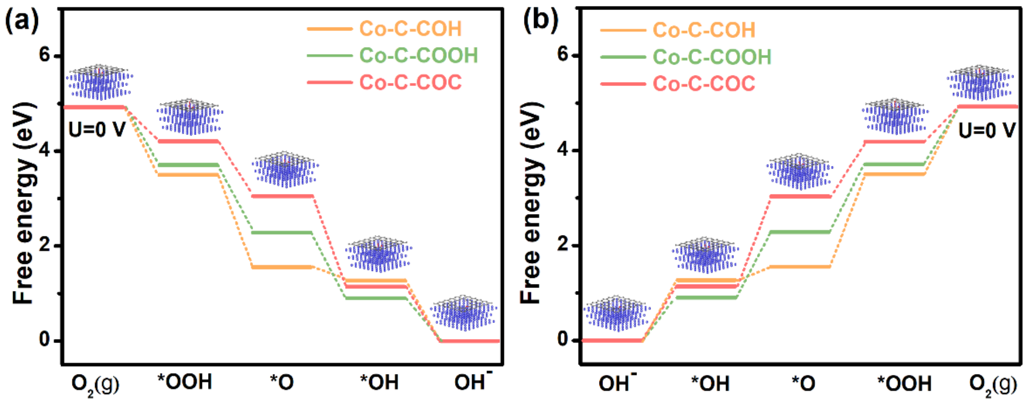

Fig. S6 Gibbs free energy polts of the (a) ORR, and (b) OER on Co-C-C=O, Co-C-COOH, and Co-C-COC at U = 0 V

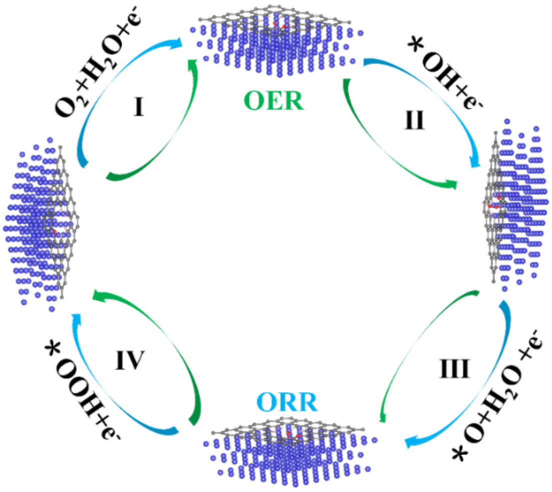

Fig. S7 ORR/OER pathways of Co-C-COC

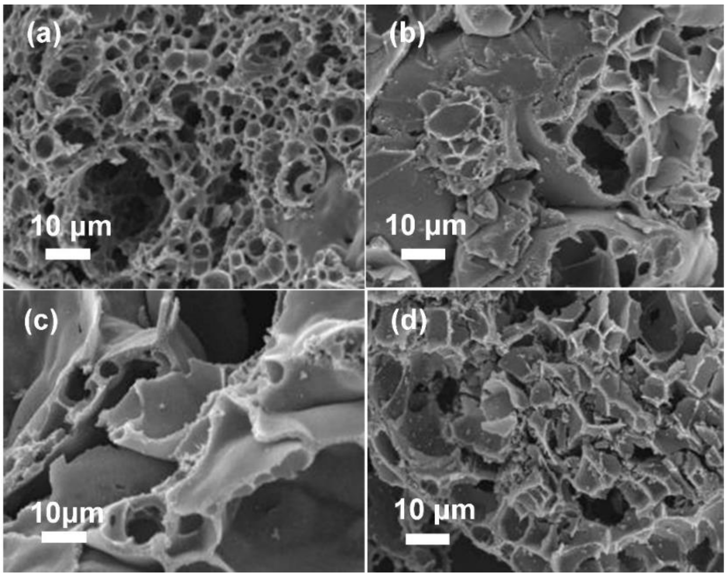

Fig. S8 SEM images of (**a**) Co@C-O-Ch, (**b**) Co@C-O-Cv, (**c**) Co@C-O-Cs, (**d**) Co@C-O-Cf

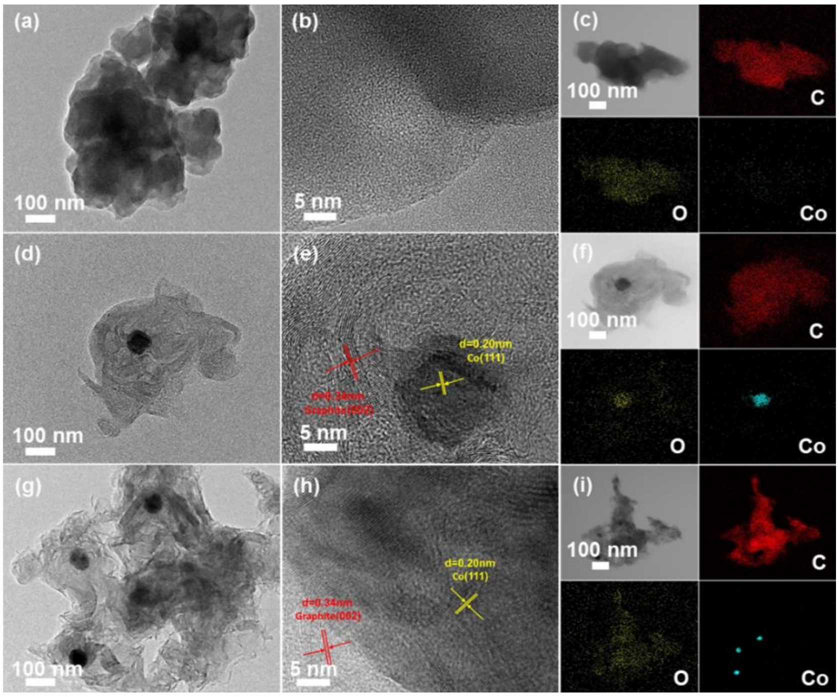

Fig. S9 (**a**) TEM, (**b**) HRTEM and (**c**) Elemental mapping images of Co@C-O-Ch; (**d**) TEM, (**e**) HRTEM and (**f**) Elemental mapping images of Co@C-O-Cv; (**g**) TEM, (**h**) HRTEM and (**i**) Elemental mapping images of Co@C-O-Cf

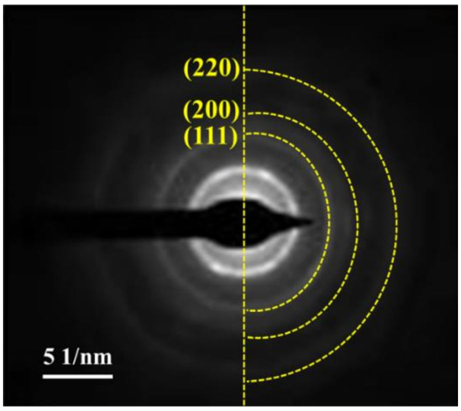

Fig. S10 SAED pattern of Co@C-O-Cs

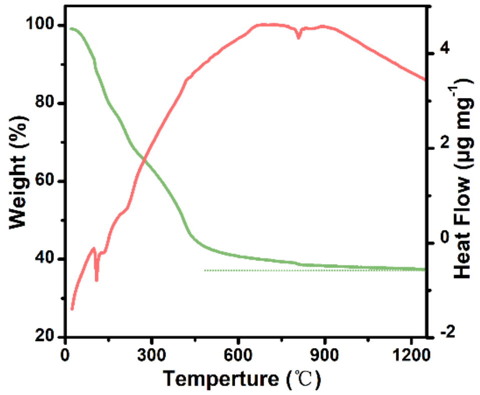

Fig. S11 TG and DSC curve of precursor Co^{2+}/CDs

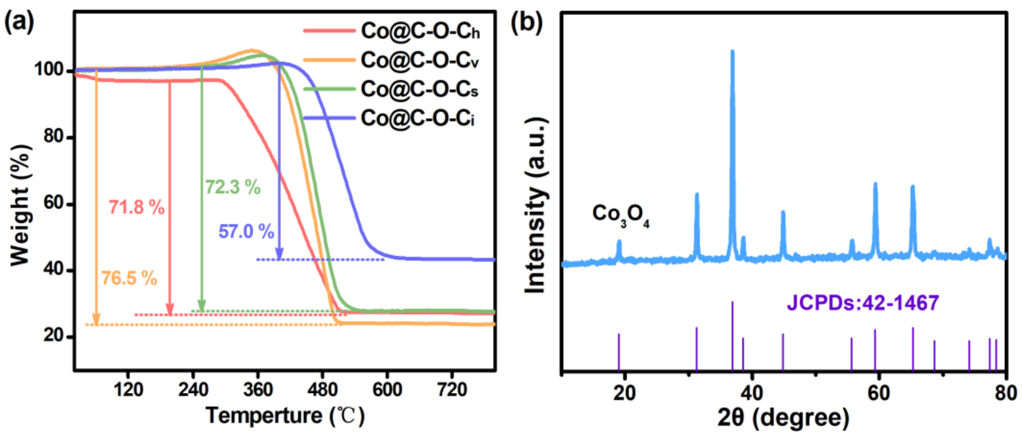

Fig. S12 (a) TG curve of Co@C-O-Ch, Co@C-O-Cv, Co@C-O-Cs, and Co@C-O-Cf; (b) XRD patterns of residual products at 1000 °C.

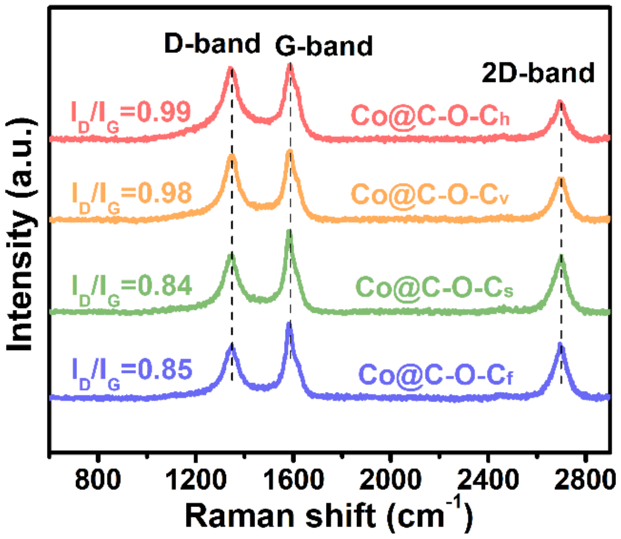

Fig. S13 Raman spectra of Co@C-O-Ch, Co@C-O-Cv, Co@C-O-Cs, and Co@C-O-Cf

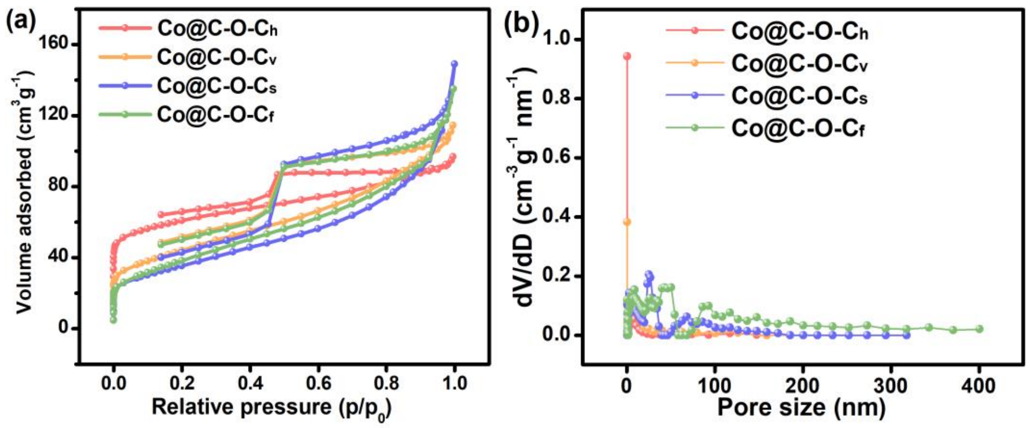

Fig. S14 (**a**) N_2 adsorption-desorption isotherms and (**b**) The pore size distribution of the Co@C-O-Ch, Co@C-O-Cv, Co@C-O-Cs, and Co@C-O-Cf

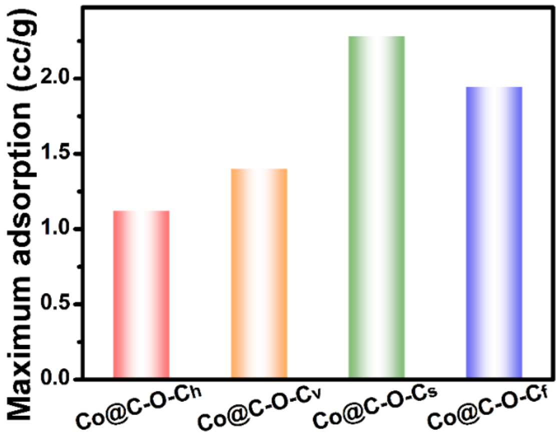

Fig. S15 The maximum oxygen adsorption of the Co@C-O-Ch, Co@C-O-Cv, Co@C-O-Cs, and Co@C-O-Cf

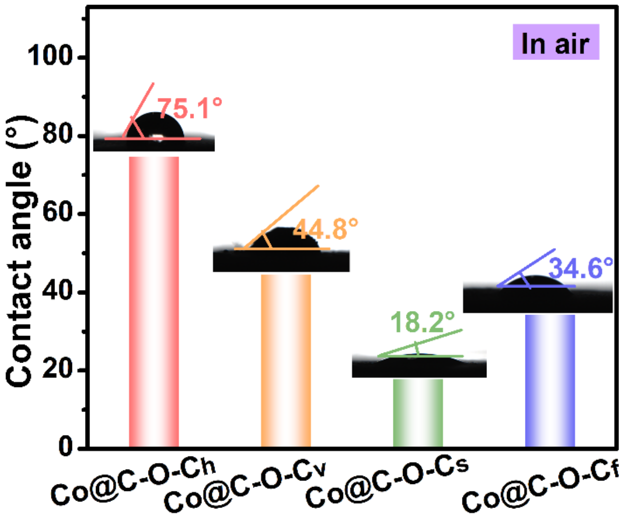

Fig. S16 Contact angle of KOH solution in air

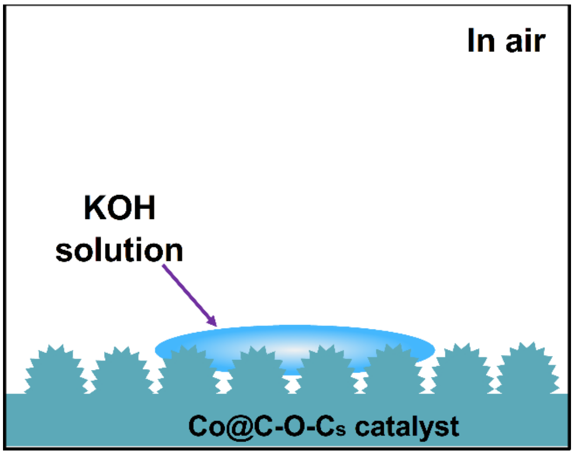

Fig. S17 Schematic aerophilicity of Co@C-O-Cs catalyst in KOH solution

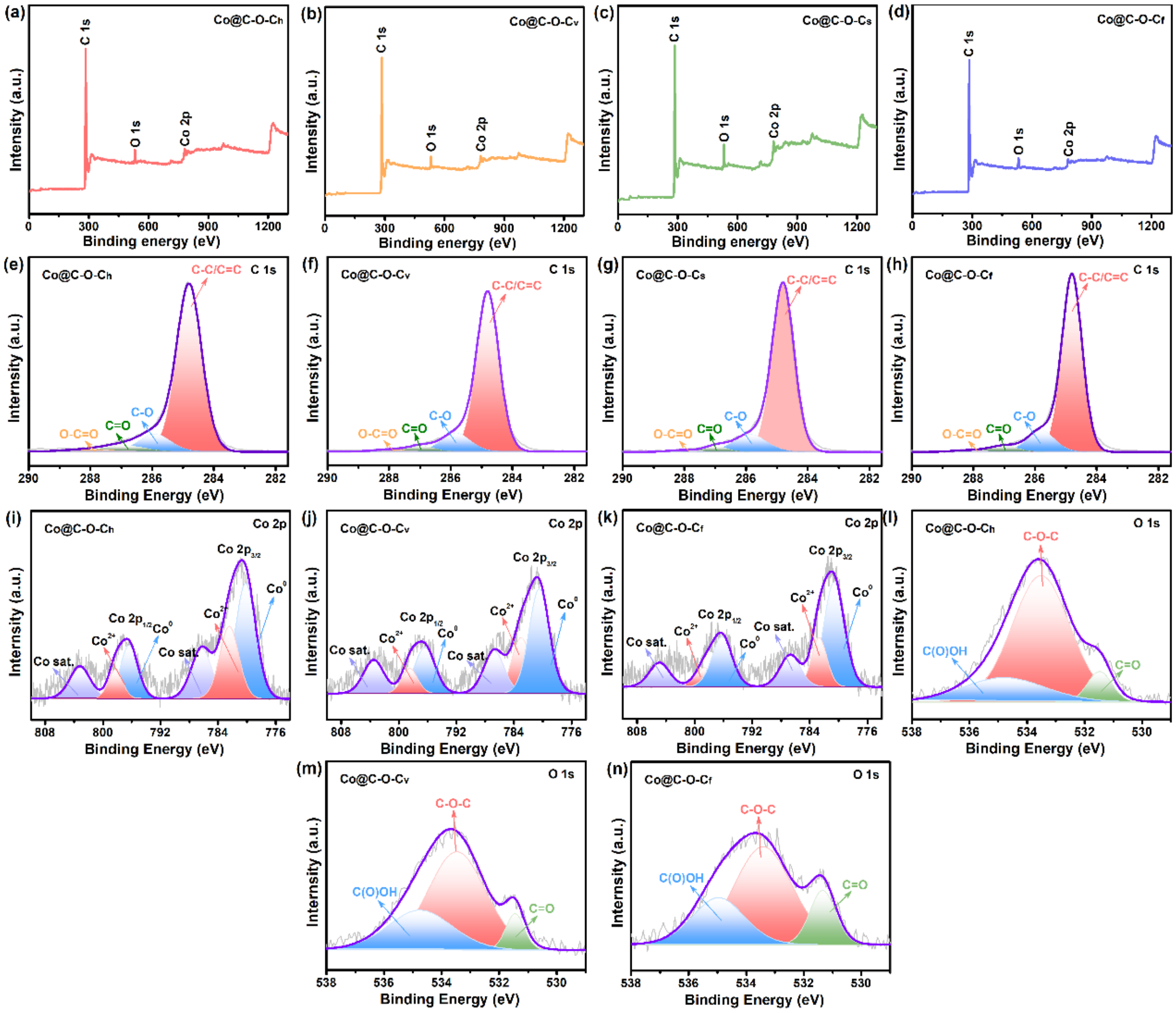

Fig. S18 (**a-d**) XPS surface survey scans and (**e-h**) C 1s spectra of Co@C-O-Ch, Co@C-O-Cv, Co@C-O-Cs, and Co@C-O-Cf; (**i-k**) Co 2p spectra and (**l-n**) O 1s spectra of Co@C-O-Ch, Co@C-O-Cv, and Co@C-O-Cf

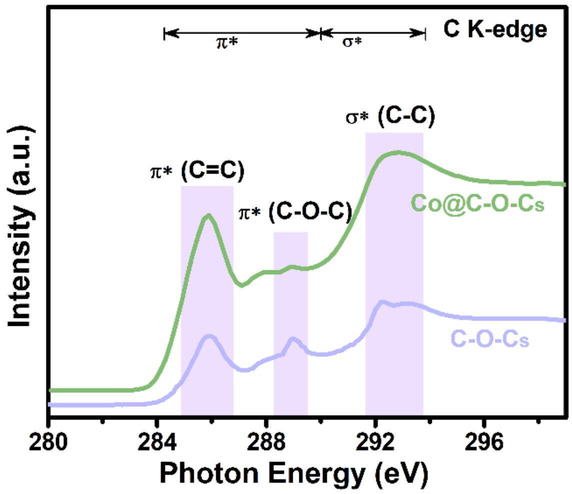

Fig. S19 C K-edge XANES spectra of Co@C-O-Cs and C-O-Cs

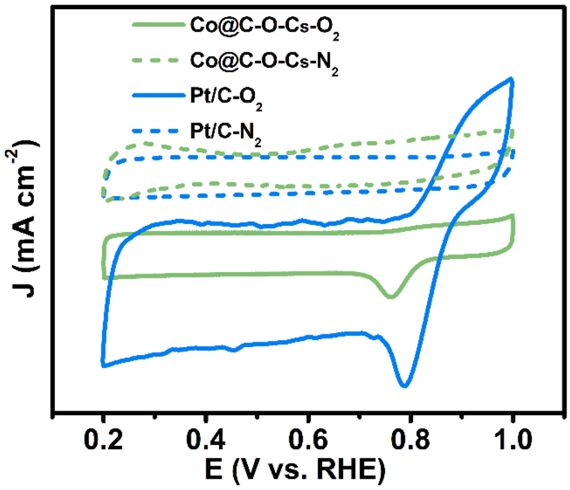

Fig. S20 CV curves of Co@C-O-Cs and commercial Pt/C in N_2-saturated and O_2-saturated 1.0 mol L^{-1} KOH with a scan rate of 10 mV s^{-1}

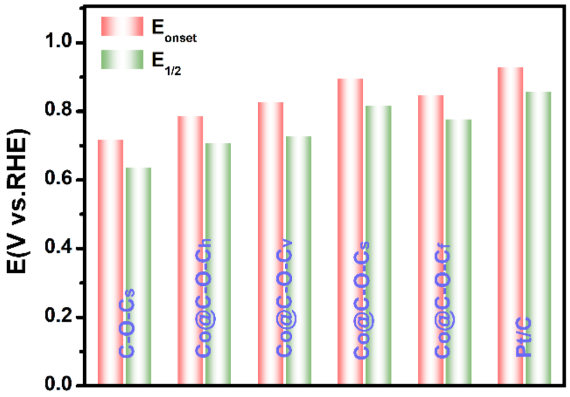

Fig. S21 Onset potentials and half-wave potentials of C-O-Cs, Co@C-O-Ch, Co@C-O-Cv, Co@C-O-Cs, Co@C-O-Cf, and Pt/C catalysts in an O_2-saturated 1.0 mol L^{-1} KOH solution at a scan speed of 5 mV·s^{-1} and a rotation rate of 1600 rpm

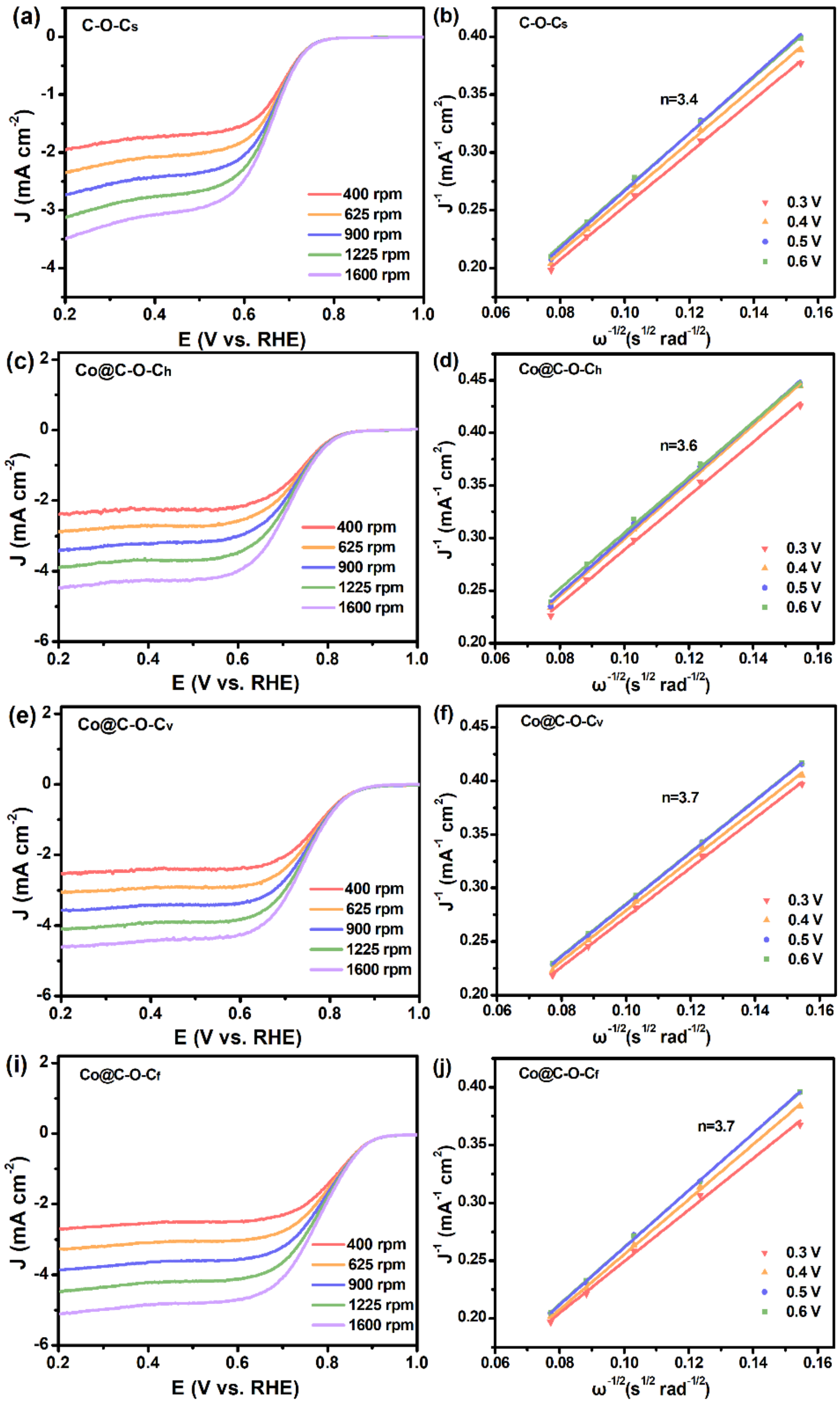

Fig. S22 LSV curves at different rotating speeds and corresponding K-L plots at different potentials: for (**a, b**) C-O-Cs; (**c, d**) Co@C-O-Ch; (**e, f**) Co@C-O-Cv; (**g, h**) Co@C-O-Cf

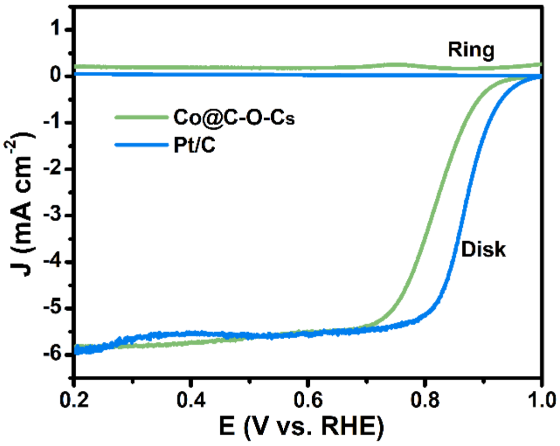

Fig. S23 Ring and disk current density of Co@C-O-Cs and Pt/C catalysts in RRDE measurements at a rotation speed of 1600 rpm.

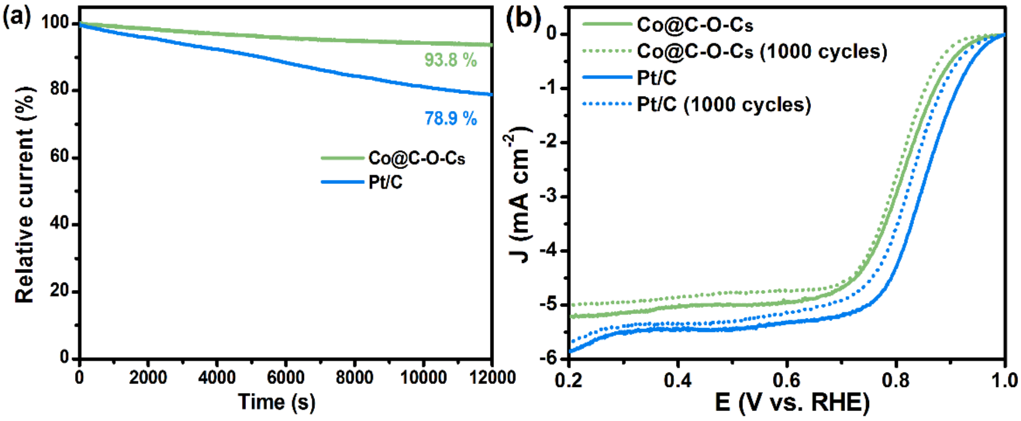

Fig. S24 (**a**) Amperometric *i-t* curves of Co@C-O-Cs and Pt/C tested in O_2-saturated 1.0 mol L^{-1} KOH solution; (**b**) ORR polarization plots for Co@C-O-Cs and Pt/C before and after 1000 cycles in O_2-saturated 1.0 mol L^{-1} KOH solution at 5 mV·s^{-1}

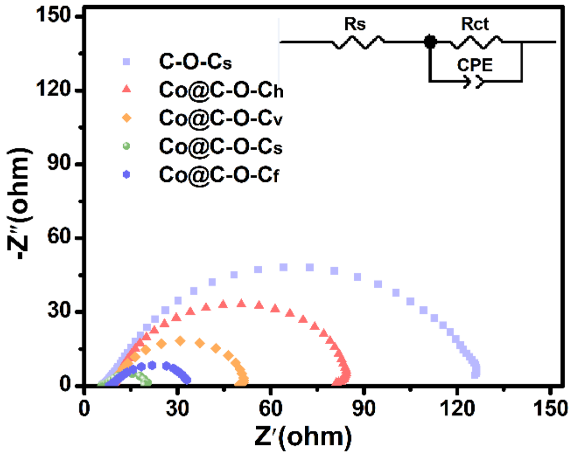

Fig. S25 Electrochemical impedance spectra (EIS) of the C-O-Cs, Co@C-O-Ch, Co@C-O-Cv, Co@C-O-Cs, and Co@C-O-Cf catalysts at an open circuit potential in 1.0 mol L^{-1} KOH solution

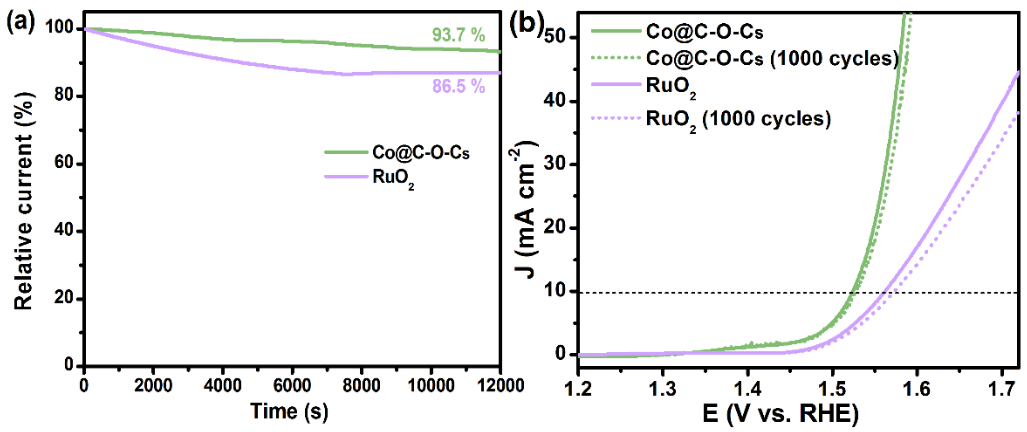

Fig. S26 (**a**) Amperometric *i-t* curves of Co@C-O-Cs and RuO_2 catalysts tested in O_2-saturated 1.0 mol L^{-1} KOH solution; (**b**) OER polarization plots for Co@C-O-Cs and RuO_2 before and after 1000 cycles in O_2-saturated 1.0 mol L^{-1} KOH solution at 5 $mV{\cdot}s^{-1}$

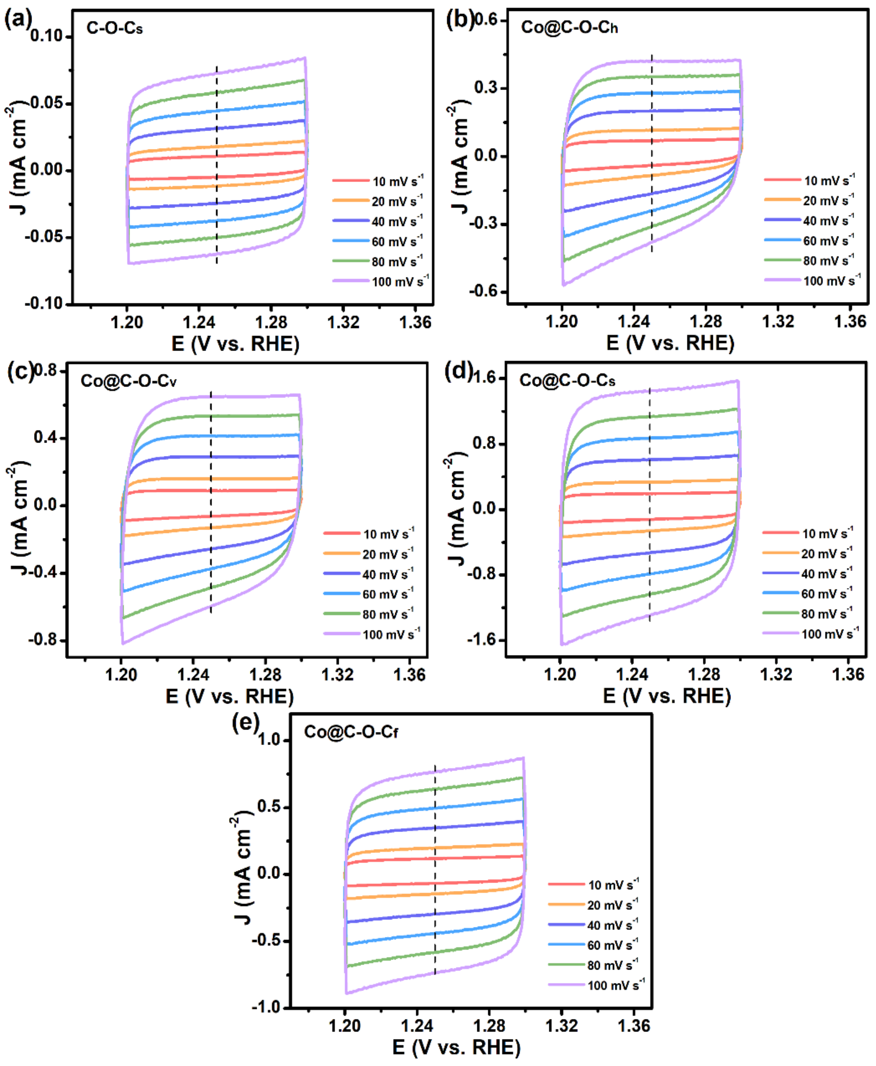

Fig. S27 Cyclic voltammograms (CVs) curves at different scan rates of (**a**) C-O-Cs; (**b**) Co@C-O-Ch; (**c**) Co@C-O-Cv; (**d**) Co@C-O-Cs; (e) Co@C-O-Cf

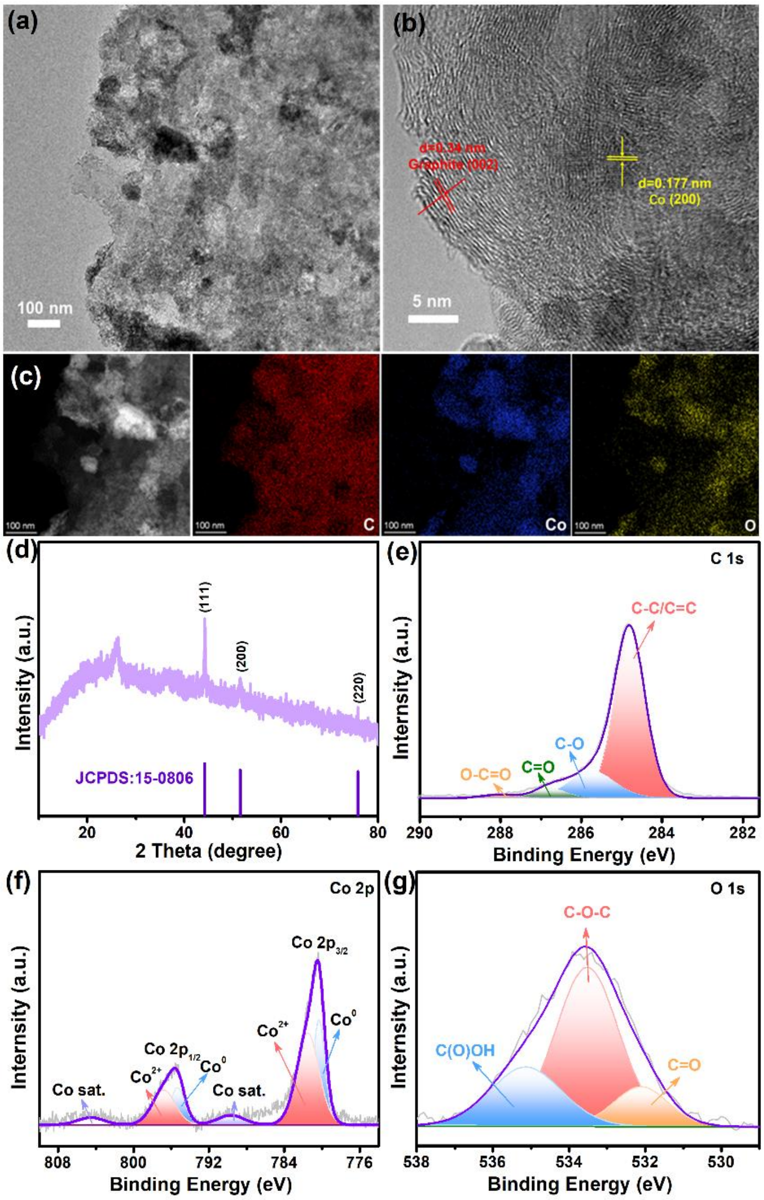

Fig. S28 (**a**) TEM, (**b**) HRTEM and (**c**) Elemental mapping images of Co@C-O-Cs after cycling; (**d**) XRD patterns of the Co@C-O-Cs after cycling; XPS spectra of (**e**) C1s, (**f**) Co 2p, (**g**) O 1s of Co@C-O-Cs after cycling

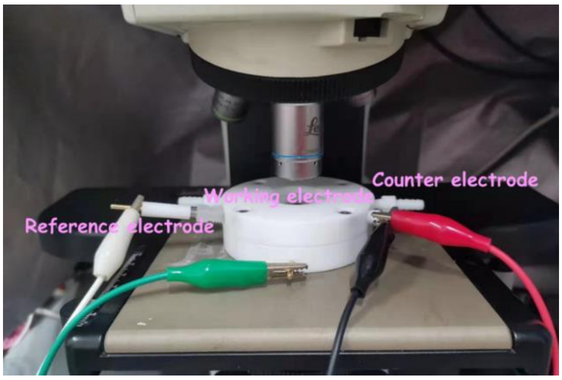

Fig. S29 Digital photograph of the chemical cell for using in in-situ Raman measurement

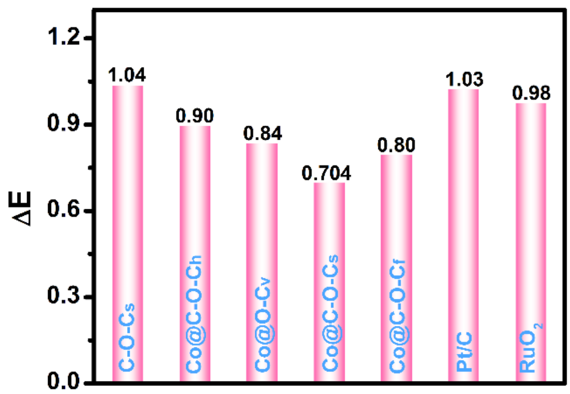

Fig. S30 ΔE values ($\Delta E = E_{j=10} - E_{1/2}$) of C-O-Cs, Co@C-O-Ch, Co@C-O-Cv, Co@C-O-Cs, Co@C-O-Cf, Pt/C, and RuO_2 catalysts

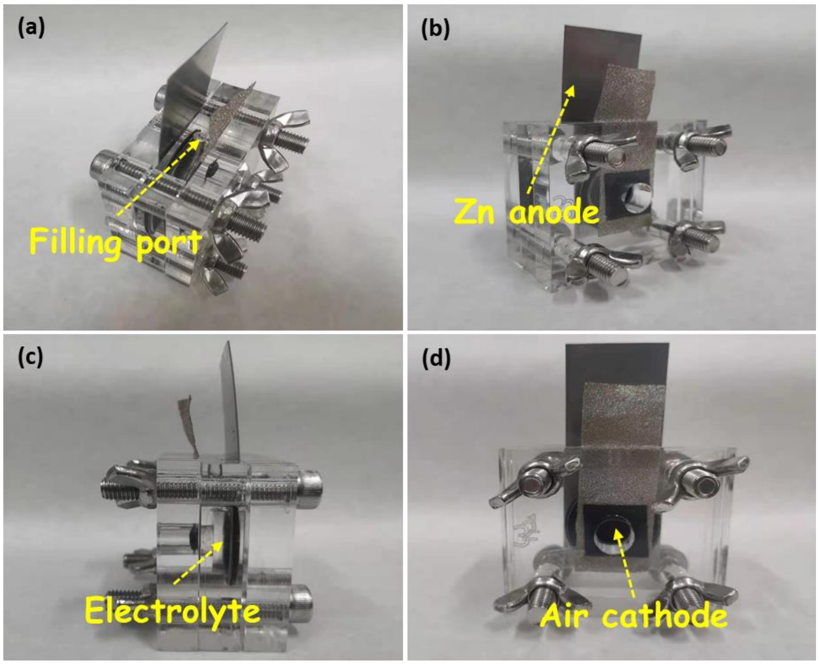

Fig. S31 Digital images of the rechargeable liquid Zn-air battery recorded from the different directions

Table S1 Comparison of ORR activity of Co@C-O-Cs with other ORR catalysts reported before

Number	Catalyst	$E_{1/2}$ / V	Electrolyte	Refs.
**1**	**Co@C-O-Cs**	**0.82**	**1.0 M KOH**	**This work**
**2**	NMGF	0.714	0.1 M KOH	[S1]
**3**	N,P-HCS	0.81	0.1 M KOH	[S2]
**4**	CMO/20N-rGO	0.79	0.1 M KOH	[S3]
**5**	Cu-N/C	0.804	0.1 M KOH	[S4]
**6**	Fe-N-CC	0.80	0.1 M KOH	[S5]
**7**	Co@Co3	0.80	0.1 M KOH	[S6]

	O4/NC-1			
**8**	N-$NiS_{1.03}$ HS	0.72	1.0 M KOH	[S7]
**9**	$Mn_3O_4/NiCo_2S_4$	0.81	0.1 M KOH	[S8]
**10**	N-$NiSe_2$/CC	0.73	0.1 M KOH	[S9]

Table S2 Comparison of OER activity of Co@C-O-Cs with other OER catalysts reported before

Number	Catalyst	Overpotential / V (10 mA cm^{-2})	Electrolyte	Refs.
**1**	**Co@C-O-Cs**	**0.294**	**1.0 M KOH**	**This work**
2	$NiCo_2O_4$/RGO-1_RGO	0.402	1.0 M KOH	[S10]
3	Fe/N/C@BMZIF	0.41	1.0 M KOH	[S11]
4	Co-NC@Al_2O_3	0.41	1.0 M KOH	[S12]
5	Y-SNi-Co-Se/CFP	0.3	0.1 M KOH	[S13]
6	NiCo/NLG-270	0.34	1.0 M KOH	[S14]
7	PPy/FeTCPP/Co	0.38	0.1 M KOH	[S15]
8	CoFe-MOF	0.351	1.0 M KOH	[S16]
9	Co-Mo_2C NPs	0.347	0.1 M KOH	[S17]
10	CoPPi nanowires	0.359	1.0 M KOH	[S18]

Table S3 Summary of the impedance fitting data for catalysts during OER process

samples	R_s (Ω)	R_{ct} (Ω)	CPE-T (mF)	CPE-P (mF)
C-O-Cs	13.60	117.10	2.95×10^{-4}	0.87434
Co@C-O-Ch	9.77	79.08	4.43×10^{-4}	0.87308
Co@C-O-Cv	9.55	43.48	4.86×10^{-4}	0.87315
Co@C-O-Cs	6.54	15.15	9.85×10^{-4}	0.84716
Co@C-O-Cf	8.87	25.73	5.84×10^{-4}	0.76717

R_s represents solution resistance in the electrolyte, R_{ct} represents charge-transfer resistance and CPE represents constant phase elements.

Table S4 Comparison of the bifunctional OER and ORR activity of Co@C-O-Cs.

Number	Catalyst	$E_{j=10}$ / V (10 mA cm^{-2})	$E_{1/2}$ / V	ΔE / V ($E_{j=10}$-$E_{1/2}$)	Refs.
**1**	**Co@C-O-Cs**	**1.524**	**0.82**	**0.704**	**This work**
2	Co-N-PC-800	1.65	0.83	0.82	[S19]

3	CoFe@N-CNWF	1.55	0.80	0.75	[S20]
4	FeCo/Se-CNT	1.65	0.9	0.75	[S21]
5	Co_3O_4@$NiCo_2O_4$	1.65	0.81	0.84	[S22]
6	Co/$ZnCo_2O_4$@NC-CNTs	1.6	0.9	0.70	[S23]
7	Co/N CCPC-3	1.631	0.832	0.799	[S24]
8	NiCo-NC	1.636	0.856	0.78	[S25]
9	Co/Co-N-C	1.640	0.882	0.758	[S26]
10	CoDNG900	1.613	0.863	0.75	[S27]

Table S5 Comparison of the power density and the performances of liquid Zn-air battery driven by Co@C-O-Cs with recently reported Zn-air batteries

**Number**	**Catalyst**	**Electrolyte**	**Power density ($mW\ cm^{-2}$)**	**Specific capacity ($mAh\ g^{-1}$)**	**Refs.**
**1**	**Co@C-O-Cs**	**6.0 M KOH + 0.2 M $Zn(Ac)_2$**	**92.1**	**700.1**	**This work**
2	Co_3O_4/NHPC	6.0 M KOH + 0.2 M $Zn(Ac)_2$	80	795	[S28]
3	Co-N-C	6.0M KOH + 0.2 M $Zn(Ac)_2$	78.75	763.2	[S29]
4	N-GCNT/FeCo-3	6.0 M KOH + 0.2 M $Zn(Ac)_2$	97.8	872.2	[S30]
5	NGM-Co	6.0 M KOH + 0.2 M $ZnCl_2$	152	749.4	[S31]
6	Co-MOF	6.0 M KOH + 0.2 M $Zn(Ac)_2$	86.2	-	[S32]
7	$NiFeO_x$/NP-C-800	6.0 M KOH + 0.2 M $Zn(Ac)_2$	82.5	688	[S33]
8	FeCo-N/C	6.0 M KOH + 0.2 M $Zn(Ac)_2$	89.9	-	[S34]
9	MCO/CNFs@NC	6.0 M KOH	75	718	[S35]
10	3C-900	6.0 M KOH + 0.2 M $ZnCl_2$	97	727	[S36]

Table S6 Comparison of the power density and the performances of flexible solid-state Zn-air battery driven by Co@C-O-Cs with recently reported Zn-air batteries

**Number**	**Catalyst**	**Electrolyte**	**Open-circuit voltage (V)**	**Power density ($mW\ cm^{-2}$)**	**Refs.**
**1**	**Co@C-O-Cs**	**18.0 M KOH + 0.2 M $Zn(Ac)_2$**	**1.434**	**59.1**	**This work**

2	Co/N@CNTs@CNMF	1.0 M KOH + 0.02 M $Zn(Ac)_2$	1.4	26.5	[S37]
3	Fe-NC SAC	18.0 M KOH + 0.2 M $Zn(Ac)_2$	1.424	45	[S38]
4	Fe-Co4N@N-C	18.0 M KOH + 0.2 M $Zn(Ac)_2$	1.34	72	[S39]
5	(Zn,Co)/NSC	18.0 M KOH + 0.2 M $Zn(Ac)_2$	1.56	15	[S40]
6	CC-AC	18.0 M KOH + 0.2 M $Zn(Ac)_2$	1.367	52.3	[S41]
7	Co-FeCo/N-G	18.0 M KOH + 0.2 M $Zn(Ac)_2$	1.419	82	[S42]
8	N, S-CC	18.0 M KOH + 0.2 M $Zn(Ac)_2$	1.247	47	[S43]
9	NGM-Co	18.0 M KOH + 0.2 M $Zn(Ac)_2$	1.439	28.2	[S31]
10	FeCo/Se-CNT	18.0 M KOH + 0.2 M $Zn(Ac)_2$	1.405	37.5	[S21]

Supplementary References

[S1] H.-F. Wang, C. Tang, Q. Zhang, Template growth of nitrogen-doped mesoporous graphene on metal oxides and its use as a metal-free bifunctional electrocatalyst for oxygen reduction and evolution reactions. Catalysis Today **301**, 25-31, (2018). https://doi.org/10.1016/j.cattod.2017.02.012

[S2] X. X. Wang, D. A. Cullen, Y.-T. Pan, S. Hwang, M. Wang et al., Nitrogen-coordinated single cobalt atom catalysts for oxygen reduction in proton exchange membrane fuel cells. Adv. Mater. **30**(11), 1706758, (2018). https://doi.org/10.1002/adma.201706758

[S3] X. He, F. Yin, S. Yuan, N. Liu, X. Huang, Hybrid spinel oxides/n-doped reduced graphene oxide as highly-active bifunctional electrocatalysts for oxygen reduction/evolution reactions. ChemElectroChem **3**(7), 1107-1115 (2016). https://doi.org/10.1002/celc.201600061

[S4] Q. Lai, Y. Zhao, J. Zhu, Y. Liang, J. He et al., Directly anchoring highly dispersed copper sites on nitrogen-doped carbon for enhanced oxygen reduction electrocatalysis. ChemElectroChem **5**(14), 1822-1826 (2018). https://doi.org/10.1002/celc.201800058

[S5] G. A. Ferrero, K. Preuss, A. Marinovic, A. B. Jorge, N. Mansor et al., Fe-n-doped carbon capsules with outstanding electrochemical performance and stability for the oxygen reduction reaction in both acid and alkaline conditions.

ACS Nano **10**(6), 5922-5932 (2016). https://doi.org/10.1021/acsnano.6b01247

[S6] A. Aijaz, J. Masa, C. Rosler, W. Xia, P. Weide et al., Co@Co_3O_4 encapsulated in carbon nanotube-grafted nitrogen-doped carbon polyhedra as an advanced bifunctional oxygen electrode. Angew. Chem. Int. Ed. **55**(12), 4087-4091 (2016). https://doi.org/10.1002/anie.201509382

[S7] J. Zhang, T. Wang, D. Xue, C. Guan, P. Xi et al., Energy-level engineered hollow N-doped $NiS_{1.03}$ for Zn-air batteries. Energy Storage Mater. **25**, 202-209 (2020). https://doi.org/10.1016/j.ensm.2019.10.014

[S8] F. Wang, G. Li, X. Meng, S. Xu, W. Ma, One-dimensional Mn_3O_4/$NiCo_2S_4$ nanocomposites as high-performance bifunctional electrocatalyst for rechargeable liquid/flexible Zn-air batteries. J. Power Sources **462**, 228162 (2020). https://doi.org/10.1016/j.jpowsour.2020.228162

[S9] S. Han, Y. Hao, Z. Guo, D. Yu, H. Huang et al., Self-supported N-doped $NiSe_2$ hierarchical porous nanoflake arrays for efficient oxygen electrocatalysis in flexible Zinc-air batteries. Chem. Eng. J. **401**, 126088 (2020). https://doi.org/10.1016/j.cej.2020.126088

[S10] K. Kumar, L. Loupias, C. Canaff, S. Morisset, S. Pronier et al., Preparation and electrochemical properties of $NiCo_2O_4$ nanospinels supported on graphene derivatives as earth-abundant oxygen bifunctional catalysts. Chem. Phys. Chem. **19**(3), 319-326 (2018). https://doi.org/10.1002/cphc.201701038

[S11] M. Wang, T. Qian, J. Zhou, C. Yan, An efficient bifunctional electrocatalyst for a zinc-air battery derived from fe/n/c and bimetallic metal-organic framework composites. ACS Appl. Mater. Interfaces **9**(6), 5213-5221 (2017). https://doi.org/10.1021/acsami.6b12197

[S12] L. Zhu, D. Zheng, Z. Wang, X. Zheng, P. Fang et al., A confinement strategy for stabilizing Zif-derived bifunctional catalysts as a benchmark cathode of flexible all-solid-state Zinc-air batteries. Adv. Mater. **30**(45), 1805268 (2018). https://doi.org/10.1002/adma.201805268

[S13] K. Ao, J. Dong, C. Fan, D. Wang, Y. Cai et al., Formation of yolk-shelled nickel-cobalt selenide dodecahedral nanocages from metal-organic frameworks for efficient hydrogen and oxygen evolution. ACS Sustain. Chem. Eng. **6**(8), 10952-10959 (2018). https://doi.org/10.1021/acssuschemeng.8b02343

[S14] X. R. Wang, J. Y. Liu, Z. W. Liu, W. C. Wang, J. Luo et al., Identifying the key role of pyridinic-N-Co bonding in synergistic electrocatalysis for reversible orr/oer. Adv. Mater. **30**(23), 1800005 (2018). https://doi.org/10.1002/adma.201800005

[S15] J. Yang, X. Wang, B. Li, L. Ma, L. Shi et al., Novel iron/cobalt-containing polypyrrole hydrogel-derived trifunctional electrocatalyst for self-powered overall water splitting. Adv. Funct. Mater. **27**(17), 1606497 (2017).

https://doi.org/10.1002/adfm.201606497

[S16] Z. Zou, T. Wang, X. Zhao, W.-J. Jiang, H. Pan et al., Expediting in-situ electrochemical activation of two-dimensional metal-organic frameworks for enhanced OER intrinsic activity by iron incorporation. ACS Catal. 7356-7364 (2019). https://doi.org/10.1021/acscatal.9b00072

[S17] M. Kim, S. Kim, D. Song, S. Oh, K. J. Chang et al., Promotion of electrochemical oxygen evolution reaction by chemical coupling of cobalt to molybdenum carbide. Appl. Catal. B **227**, 340-348 (2018). https://doi.org/10.1016/j.apcatb.2018.01.051

[S18] H. Du, W. Ai, Z. L. Zhao, Y. Chen, X. Xu et al., Engineering morphologies of cobalt pyrophosphates nanostructures toward greatly enhanced electrocatalytic performance of oxygen evolution reaction. Small **14**(31), 1801068 (2018). https://doi.org/10.1002/smll.201801068

[S19] W. Zhou, Y. Li, L. Zheng, J. Liu, R. Tang et al., Three-dimensional mof-derived co and n co-doped porous carbon bifunctional catalyst for the Zn-air battery. CrysTengComm **23**(28), 4930-4937 (2021). https://doi.org/10.1039/d1ce00761k

[S20] L. Zhang, Y. Zhu, Z. Nie, Z. Li, Y. Ye et al., Co/MoC nanoparticles embedded in carbon nanoboxes as robust trifunctional electrocatalysts for a Zn-air battery and water electrocatalysis. ACS Nano **15**(8), 13399-13414 (2021). https://doi.org/10.1021/acsnano.1c03766

[S21] H. Zhang, M. Zhao, H. Liu, S. Shi, Z. Wang et al., Ultrastable feco bifunctional electrocatalyst on Se-doped cnts for liquid and flexible all-solid-state rechargeable Zn-air batteries. Nano Lett. **21**(5), 2255-2264 (2021). https://doi.org/10.1021/acs.nanolett.1c00077

[S22] N.-F. Yu, W. Huang, K.-L. Bao, H. Chen, K. Hu et al., Co_3O_4@$NiCo_2O_4$ double-shelled nanocages with hierarchical hollow structure and oxygen vacancies as efficient bifunctional electrocatalysts for rechargeable zn-air batteries. Dalton Trans. **50**(6), 2093-2101 (2021). https://doi.org/10.1039/d0dt03971c

[S23] L. Yan, Z. Xu, W. Hu, J. Ning, Y. Zhong et al., Formation of sandwiched leaf-like CNTs-Co/$ZnCo_2O_4$@NC-CNTs nanohybrids for high-power-density rechargeable zn-air batteries. Nano Energy **82**, 105710 (2021). https://doi.org/10.1016/j.nanoen.2020.105710

[S24] L. Yan, H. Wang, J. Shen, J. Ning, Y. Zhong et al., Formation of mesoporous Co/CoS/metal-N-C@S, N-codoped hairy carbon polyhedrons as an efficient trifunctional electrocatalyst for Zn-air batteries and water splitting. Chem. Eng. J. **403**, 126385 (2021). https://doi.org/10.1016/j.cej.2020.126385

[S25] F. Wang, Y. Xu, Y. Wang, Z. Liang, R. Zhang et al., Space-confined construction of two-dimensional nitrogen-doped carbon with encapsulated bimetallic nanoparticles as oxygen electrocatalysts. Chem. Commun. **57**(66), 8190-8193

(2021). https://doi.org/10.1039/d1cc02591k

[S26] D. Wang, P. Yang, H. Xu, J. Ma, L. Du et al., The dual-nitrogen-source strategy to modulate a bifunctional hybrid Co/Co-N-C catalyst in the reversible air cathode for Zn-air batteries. J. Power Sources **485**, 229339 (2021). https://doi.org/10.1016/j.jpowsour.2020.229339

[S27] A. Wang, C. Zhao, M. Yu, W. Wang, Trifunctional co nanoparticle confined in defect-rich nitrogen-doped graphene for rechargeable Zn-air battery with a long lifetime. Appl. Catal. B **281**, 119514 (2021). https://doi.org/10.1016/j.apcatb.2020.119514

[S28] J. Guan, Z. Zhang, J. Ji, M. Dou, F. Wang, Hydrothermal synthesis of highly dispersed Co_3O_4 nanoparticles on biomass-derived nitrogen-doped hierarchically porous carbon networks as an efficient bifunctional electrocatalyst for oxygen reduction and evolution reactions. ACS Appl. Mater. Interfaces **9**(36), 30662-30669 (2017). https://doi.org/10.1021/acsami.7b08533

[S29] H. Liu, M.-Q. Wang, Z.-Y. Chen, H. Chen, M.-W. Xu et al., Design and synthesis of Co-N-C porous catalyst derived from metal organic complexes for highly effective ORR. Dalton Trans. **46**(45), 15646-15650 (2017). https://doi.org/10.1039/c7dt03279j

[S30] C.-Y. Su, H. Cheng, W. Li, Z.-Q. Liu, N. Li et al., Atomic modulation of FeCo-nitrogen-carbon bifunctional oxygen electrodes for rechargeable and flexible all-solid-state zinc-air battery. Adv. Energy Mater. **7**(13), 1602420 (2017). https://doi.org/10.1002/aenm.201602420

[S31] C. Tang, B. Wang, H. F. Wang, Q. Zhang, Defect engineering toward atomic Co-N_x-C in hierarchical graphene for rechargeable flexible solid Zn-air batteries. Adv. Mater. **29**(37), 1703185 (2017). https://doi.org/10.1002/adma.201703185

[S32] G. Chen, J. Zhang, F. Wang, L. Wang, Z. Liao et al., Cobalt-based metal-organic framework nanoarrays as bifunctional oxygen electrocatalysts for rechargeable Zn-air batteries. Chem. Eur. J. **24**(69), 18413-18418 (2018). https://doi.org/10.1002/chem.201804339

[S33] X. Chen, L. Wei, Y. Wang, S. Zhai, Z. Chen et al., Milk powder-derived bifunctional oxygen electrocatalysts for rechargeable Zn-air battery. Energy Storage Mater. **11**, 134-143 (2018). https://doi.org/10.1016/j.ensm.2017.10.011

[S34] X.-W. Gao, J. Yang, K. Song, W.-B. Luo, S.-X. Dou et al., Robust feco nanoparticles embedded in a n doped porous carbon framework for high oxygen conversion catalytic activity in alkaline and acidic media. J. Mater. Chem. A **6**(46), 23445-23456 (2018). https://doi.org/10.1039/c8ta06382f

[S35] T. T. Gebremariam, F. Chen, Q. Wang, J. Wang, Y. Liu et al., Bimetallic Mn-Co oxide nanoparticles anchored on carbon nanofibers wrapped in nitrogen-doped carbon for application in Zn-air batteries and supercapacitors. ACS Appl.

Energy Mater. **1**(4), 1612-1625 (2018). https://doi.org/10.1021/acsaem.8b00067

[S36] T. Li, Y. Lu, S. Zhao, Z.-D. Gao, Y.-Y. Song, Co_3O_4-doped Co/CoFe nanoparticles encapsulated in carbon shells as bifunctional electrocatalysts for rechargeable Zn-air batteries. J. Mater. Chem. A **6**(8), 3730-3737 (2018). https://doi.org/10.1039/c7ta11171a

[S37] T. Liu, J. Mou, Z. Wu, C. Lv, J. Huang et al., A facile and scalable strategy for fabrication of superior bifunctional freestanding air electrodes for flexible zinc–air batteries. Adv. Funct. Mater. **30**(36), 2003407 (2020). https://doi.org/10.1002/adfm.202003407

[S38] C. Du, Y. Gao, J. Wang, W. Chen, A new strategy for engineering a hierarchical porous carbon-anchored fe single-atom electrocatalyst and the insights into its bifunctional catalysis for flexible rechargeable Zn-air batteries. J. Mater. Chem. A **8**(19), 9981-9990 (2020). https://doi.org/10.1039/d0ta03457f

[S39] Q. Xu, H. Jiang, Y. Li, D. Liang, Y. Hu et al., In-situ enriching active sites on co-doped Ffe-Co_4N@N-C nanosheet array as air cathode for flexible rechargeable Zn-air batteries. Appl. Catal. B **256**, 117893 (2019). https://doi.org/10.1016/j.apcatb.2019.117893

[S40] D. Liu, B. Wang, H. Li, S. Huang, M. Liu et al., Distinguished Zn, Co-N_x-C-S_y active sites confined in dentric carbon for highly efficient oxygen reduction reaction and flexible Zn-air batteries. Nano Energy **58**, 277-283 (2019). https://doi.org/10.1016/j.nanoen.2019.01.011

[S41] K. Kordek, L. Jiang, K. Fan, Z. Zhu, L. Xu et al., Two-step activated carbon cloth with oxygen-rich functional groups as a high-performance additive-free air electrode for flexible zinc-air batteries. Adv. Energy Mater. **9**(4), 1802936 (2019). https://doi.org/10.1002/aenm.201802936

[S42] T. Wang, X. Guo, J. Zhang, W. Xiao, P. Xi et al., Electronic structure modulation of NiS_2 by transition metal doping for accelerating the hydrogen evolution reaction. J. Mater. Chem. A **7**(9), 4971-4976 (2019). https://doi.org/10.1039/c8ta11286j

[S43] Z. Zhao, Z. Yuan, Z. Fang, J. Jian, J. Li et al., In situ activating strategy to significantly boost oxygen electrocatalysis of commercial carbon cloth for flexible and rechargeable Zn-air batteries. Adv. Sci. **5**(12), 1800760 (2018). https://doi.org/10.1002/advs.201800760
